# Paneth-like transition drives resistance to dual targeting of KRAS and EGFR in colorectal cancer

**DOI:** 10.1016/j.ccell.2025.10.010

**Published:** 2025-11-13

**Authors:** Yuetong Zhang, Jiaying Chen, Yong She, Zhaoyuan Fang, Yaxin Zhang, Danyun Ruan, Wenjun Guo, Jianping Liao, Weiping Zhou, Jianpei Lao, Weicheng Fang, Xingyan Pan, Wenfei Kang, Zifeng Wang, Yuanzhong Wu, Rong Deng, Lin Tian, Liqin Wang, Huilin Huang, Jian Zheng, Yan Yan, Hezhe Lu, Ruiping Wang, Rona Yaeger, Qi Zhao, Wenting Liao, Feng Wang, Yijun Gao

**Affiliations:** 1 State Key Laboratory of Oncology in South China, Guangdong Provincial Clinical Research Center for Cancer, Sun Yat-sen University Cancer Center, Guangzhou 510060, China; 2 Department of Medical Oncology, Sun Yat-sen University Cancer Center, State Key Laboratory of Oncology in South China, Collaborative Innovation Center for Cancer Medicine, Sun Yat-sen University, Guangzhou 510060, P.R. China; 3 Research Unit of Precision Diagnosis and Treatment for Gastrointestinal Cancer, Chinese Academy of Medical Sciences, Guangzhou 510060, P.R. China; 4 Department of Clinical Research, Sun Yat-sen University Cancer Center, State Key Laboratory of Oncology in South China, Guangdong Provincial Clinical Research Center for Cancer, Sun Yat-sen University, Guangzhou, China; 5 Bioinformatics Platform, Department of Experimental Research, Sun Yat-sen University Cancer Center, State Key Laboratory of Oncology in South China, Collaborative Innovation Center for Cancer Medicine, Guangzhou, P.R. China; 6 Department of Colorectal Surgery and Oncology of the Second Affiliated Hospital, and Centre of Biomedical Systems and Informatics of Zhejiang University-University of Edinburgh Institute (ZJU-UoE Institute), Zhejiang University School of Medicine, Zhejiang University, Hangzhou 310058, China; 7 State Key Laboratory of Organ Regeneration and Reconstruction, Institute of Zoology, Institute for Stem Cell and Regeneration, Chinese Academy of Sciences, Beijing, China; 8 Molecular Cytology Core Facility, Memorial Sloan Kettering Cancer Center, New York, NY, USA; 9 College of Biomedicine and Health and College of Life Science and Technology, Hubei Hongshan Laboratory, Huazhong Agricultural University, Wuhan, Hubei 430070, China; 10 Department of Medicine, Memorial Sloan Kettering Cancer Center, New York, NY, USA; 11 These authors contributed equally; 12 Lead contact

## Abstract

While dual KRAS and epidermal growth factor receptor (EGFR) inhibition shows promise in treating *KRAS*-mutant colorectal cancer (CRC), resistance remains a major challenge. Using genetically engineered mouse models, patient-derived organoids and xenografts, as well as clinical specimens, we discover that colorectal tumors surviving combined KRAS and EGFR inhibition acquire a Paneth-like cell state—a secretory lineage typically confined to the intestinal crypt. Lineage tracing reveals that CRC cells evade dual therapy by transitioning into a Paneth-like state. Through integrated transcriptomic analysis and CRISPR genetic screening, we identify SMAD1 as a key regulator of this lineage plasticity, promoting resistance by directly activating FGFR3. Genetic or pharmacological inhibition of FGFR3 prevents the Paneth-like transition, restores drug sensitivity, and synergizes with KRAS-EGFR inhibition across multiple preclinical models. These findings reveal that the SMAD1-FGFR3 axis triggers Paneth-like plasticity to drive KRAS-EGFR dual therapy resistance in CRC and highlight FGFR3 blockade as a promising strategy to overcome plasticity-driven drug tolerance.

## In brief

Zhang et al. show that CRC cells adopt a Paneth-like state to evade dual KRAS and EGFR inhibition. They identify a SMAD1-FGFR3 axis that drives lineage plasticity and MAPK reactivation, conferring resistance. FGFR3 co-targeting blocks this transition, offering a strategy to improve the durability of KRAS-EGFR combination therapies.

## Graphical abstract

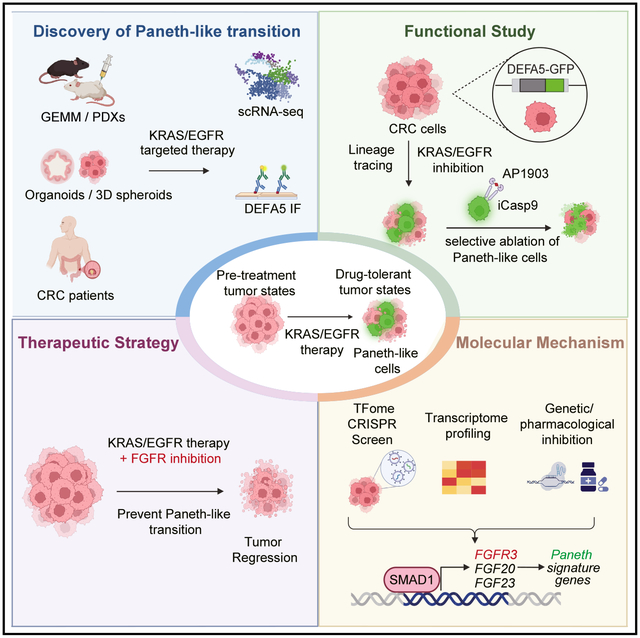

## INTRODUCTION

*KRAS* is one of the most commonly mutated oncogenes in human cancer, with mutations observed in up to 50% of colorectal cancer (CRC) cases.^1,2^ *KRAS*-mutant CRC has long posed a therapeutic challenge, with poor prognosis due to limited therapeutic options.^3^ However, recent breakthroughs with KRAS G12C inhibitors (e.g., sotorasib and adagrasib) in combination with epidermal growth factor receptor (EGFR) antibodies have transformed therapeutic strategies for *KRAS G12C*-mutant CRC. This combination therapy achieves objective response rates of 34%–46% in patients with *KRAS G12C*-mutant CRC, prompting accelerated Food and Drug Administration approval for this regimen.^4–7^ Success with KRAS G12C inhibitors in both non-small cell lung cancer (NSCLC) and CRC has spurred the development of innovative KRAS inhibitors targeting other common KRAS oncogenic mutations.^8–11^ For instance, MRTX1133—a KRAS G12D inhibitor—demonstrates potent anti-tumor effects in preclinical NSCLC and pancreatic ductal adenocarcinoma (PDAC) models^12–15^ and in CRC models when combined with EGFR antibody.^13,16^ Thus, MRTX1133 serves as a valuable tool compound for studying KRAS G12D-driven biology and therapeutic vulnerabilities.

While KRAS and EGFR dual targeting holds promise in *KRAS-*mutant CRC, responses are often short-lived; most patients relapse within months due to acquired resistance. Recent studies have identified several mechanisms of resistance to this combination, including secondary *KRAS* mutations, alterations in genes upstream or downstream of KRAS, and alterations in other RAS homologs.^7,17,18^ Moreover, about a third of relapsed tumors lack detectable genomic alterations,^18,19^ suggesting that non-genetic mechanisms also play a significant role in resistance to KRAS-EGFR dual therapy.

Notably, multiple resistance mutations often emerge in relapsed tumors within the same patient,^7,17,18^ which poses challenges to targeting heterogenous resistance mechanisms at the time of progressing disease. In this context, targeting minimal residual disease (MRD) before these genetic resistance alterations emerge could be key to delay the development of resistance. However, our current understanding of MRD state after targeted therapy remains challenging due to the difficulty of obtaining repeated biopsies. Tracking the evolution of cancer cell state following KRAS and EGFR combination therapy through *in vivo* genetically engineered mouse models (GEMMs) and xenograft models that recapitulate human CRC is urgently needed to fill up this knowledge gap.

Intestinal cells are well characterized with high plasticity, enabling them to de-differentiate into a stem-like state and regenerate the intestinal crypts during tissue injury and stress.^20–23^ Recent studies suggest that CRC cells also exhibit remarkable cellular plasticity to survive treatment, transitioning into alternative cell states under therapeutic pressure. CRC cells reprogram into a fetal progenitor-like state and further differentiate into squamous and neuroendocrine lineages to evade chemotherapy.^24^ In another study using RAS-RAF wild-type CRC patient-derived xenograft (PDX) models, upregulation of Paneth cell lineage markers is observed in surviving CRC cells following EGFR antibody treatment.^25^ These studies indicate that CRC cells are able to alter their differentiation state in response to the selective pressure imposed by different therapies. This is corroborated by studies interrogating cancer cell states driving resistance to KRAS-targeted therapy in *KRAS-*mutant NSCLC and PDAC. Differentiation into alveolar type 1 (AT1)-like,^26^ squamous,^27,28^ as well as mucinous states^29^ has been implicated to contribute to KRAS inhibitor tolerance in lung adenocarcinoma, whereas transition into a classical state is observed in PDAC cells surviving KRAS-targeted therapy.^30,31^ While these studies demonstrate that tumor cells from lung and pancreas lineage adopt distinct routes of *trans*-differentiation to evade KRAS inhibition, how CRC cells reprogram their cellular states in response to KRAS-EGFR combination therapy remains an unresolved question. Further, although heterogenous cell states have been associated with resistance, the underlying molecular mechanisms and key transcription factor driving lineage switches under therapeutic pressure have yet to be defined.

In this study, we investigate the impact of combined KRAS and EGFR inhibition on cell states in *KRAS* mutant-driven CRC models including GEMMs, human CRC organoids, and PDX models. Furthermore, we conduct mechanistic studies into Paneth-like transition through lineage tracing, transcriptomic analysis, and functional screens and identify targetable molecular drivers to overcome lineage plasticity-driven therapy resistance.

## RESULTS

### Enrichment of Paneth-like cell state in residual CRC lesions following KRAS-EGFR inhibition

**Figure 1.**
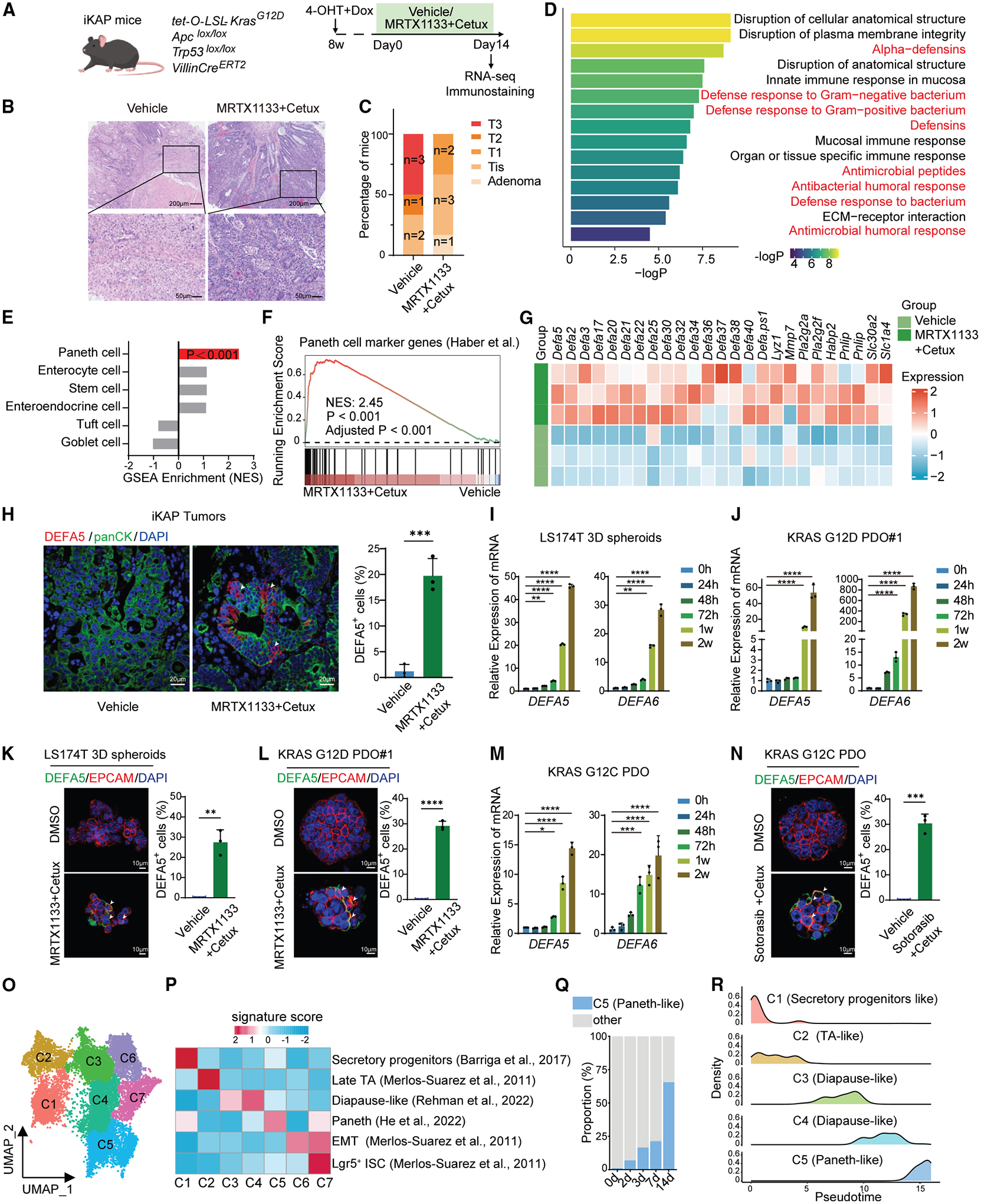
Enrichment of the Paneth-like cell state in residual CRC lesions following KRAS-EGFR inhibition (A) Schematic of the iKAP CRC model treated with vehicle or MRTX1133 + cetuximab for 2 weeks (*n* = 6). (B and C) Representative H&E images (B) and T classification quantification (C) in iKAP tumors treated with MRTX1133 + cetuximab or vehicle. Scale bars: 200 μm (overview, top) and 50 μm (magnified, bottom). (D) Bar plot depicting the pathway enrichment analysis for upregulated genes in MRTX1133 + cetuximab-treated iKAP tumors. Pathways associated with “Paneth cells” are highlighted in red. (E) Gene set enrichment analysis (GSEA) showing alterations of intestinal epithelial cell-type-specific signatures in MRTX1133 and cetuximab-treated tumors relative to vehicle-treated tumors. Gene sets that were upregulated or downregulated are represented by normalized enrichment scores (NES) on the *x* axis. Significant hits (adjusted *p* value < 0.05) are indicated in red. (F) GSEA plot denoting that MRTX1133 + cetuximab treatment promoted the expression of Paneth cell signature genes. (G) Heatmap of Paneth cell marker genes in iKAP tumors. Expression values represented as *Z* score of log2-transformed TPM. (H) Immunofluorescence staining and quantification of DEFA5, panCK, and DAPI (representative of *n* = 3 mice). Scale bars: 20 μm. (I, J, and M) Quantitative reverse-transcription PCR (RT-qPCR) analysis of Paneth cell marker genes *DEFA5* and *DEFA6*, in LS174T 3D spheroids (I), KRAS G12D PDO#1 (J), and KRAS G12C PDO (M) following time-course treatment with KRASi (100 nM) + cetuximab (25 nM). Expression levels were normalized to the reference gene *GAPDH*. Data are presented as mean ± SD (*n* = 3). (K, L, and N) Representative images and quantification of immunofluorescence staining of DEFA5, EPCAM, and DAPI in LS174T 3D spheroids (K), KRAS G12D PDO#1 (L), and KRAS G12C PDO (N) treated with KRASi (100 nM) + cetuximab (25 nM) (*n* = 3 fields). Scale bars: 10 μm. (O) UMAP embedding and visualization of seven cell states (C1 to C7). (P) Heatmap of UCell signature enrichment scores averaged in each cell state. (Q and R) Temporal dynamics of the C5 cell state. (Q) Expansion of the C5 cell state along the five experimental sampling time points during drug treatment. (R) Density distribution of cell states along the Monocle pseudotime. Data are represented as mean ± SD (H–N). Statistical analyses were conducted using permutation testing (with multiple hypothesis correction) for GSEA (E and F), two-tailed *t* test (H, K, L, and N), or one-way ANOVA (I, J, and M). *p < 0.05, **p < 0.01, ***p < 0.001, ****p < 0.0001. See also Figure S1.

To investigate the response of mutant *KRAS G12D*-driven CRC tumors to combined KRAS and EGFR inhibition, we leveraged the iKAP (*tet-O-LSL-Kras*^*G12D*^, *Apc*^*L/L*^, *Trp53*^*L/L*^, *Villin*^*CreERT2*^) GEMM model.^32^ Autochthonous CRC tumors were induced in iKAP mice via enema administration of 4-hydroxytamoxifen and doxycycline water at 8 weeks of age. The mice were then treated with the KRAS G12D inhibitor MRTX1133 and EGFR antibody cetuximab or vehicle for 2 weeks (Figure 1A). The combined therapy dramatically suppressed tumor progression and improved tumor histology (Figures 1B, 1C, and S1A). Consistently, inhibition of ERK signaling and tumor proliferation (Ki-67), as well as increased apoptosis, was observed in the residual tumors following MRTX1133 and cetuximab treatment (Figures S1B–S1E). Transcriptomic analysis of residual tumors identified enriched pathways characteristic of Paneth cells, including defensins and antimicrobial peptides (Figure 1D). We further conducted gene set enrichment analysis utilizing cell-type-specific signatures from a published RNA sequencing (RNA-seq) study^32^ and discovered that the Paneth cell signature was the most significantly enriched epithelial cell type following combination treatment (Figures 1E, 1F, and S1F). Paneth cells are a type of secretory epithelial cells that typically reside at the base of small intestinal crypts.^33^ Previous studies have well documented the essential role of Paneth cells in maintaining intestinal tissue homeostasis by secreting antimicrobial peptides and providing niche signaling to support LGR5^+^ intestinal stem cells.^34^ Comparative analysis revealed that residual tumor cells shared gene expression signatures with intestinal Paneth cells but differed from Paneth-like cells in the colon^35^(Figure S1G), as well as *Reg4*^+^ deep crypt secretory cells (Figure S1H), which are functionally analogous to Paneth cells in the murine colon.^36,37^ Evaluation of Paneth cell lineage-specific genes revealed upregulation of multiple defensins (e.g., *Defa5* and *Defa17*) and *Lyz1* in tumors treated with MRTX1133 and cetuximab (Figure 1G). Immunofluorescence staining of DEFA5 and LYZ further validated robust induction of Paneth-like cells by MRTX1133 and cetuximab treatment in iKAP CRC tumors (Figures 1H and S1I). These results indicate that the Paneth-like cell state is enriched in iKAP tumors surviving combined KRAS and EGFR inhibition.

To determine whether Paneth-like cells could be enriched in human *KRAS*-mutant CRC cells following KRAS-EGFR inhibition, we modeled drug responses using colospheres of the *KRAS G12D*-mutant CRC cell line LS174T and two patient-derived organoids (PDOs). Combined KRAS-EGFR inhibition significantly increased the expression of Paneth cell-specific markers *DEFA5* and *DEFA6*, with most markers upregulated by 72 h and progressively elevated for up to 2 weeks (Figures 1I, 1J, and S1J). Immunofluorescence analysis further demonstrated a striking increase of DEFA5^+^ cells in tumor spheroids post treatment (Figures 1K, 1L, and S1K). To confirm that our finding is not specific to the *KRAS G12D* allele, we treated two *KRAS G12C* CRC cell lines (SW1463 and RW7213) and a PDO with KRAS G12C inhibitor sotorasib and cetuximab. Similarly, Paneth-like cell enrichment was observed in all models following combined inhibition (Figures 1M, 1N, and S1L–S1O).

To delineate the dynamic cell-fate transitions underlying the emergence of the Paneth-like state, we conducted time-resolved single-cell RNA sequencing (scRNA-seq) on a *KRAS G12D* PDO treated with MRTX1133 and cetuximab for 0, 2, 3, 7, and 14 days. We scored the CRC PDO cells for the published Paneth-like state signatures^38,39^ and observed a gradual increase in Paneth-like gene module activity over time (Figure S1P), supporting a stepwise transition during treatment. scRNA-seq analysis identified seven transcriptionally distinct epithelial populations (C1–C7), with cluster C5 enriched for Paneth cell lineage markers and expanding progressively with treatment (Figures 1O–1Q). Trajectory analysis further revealed that Paneth-like cells emerged from a diapause-like drug-tolerant persister (DTP) intermediate (clusters C3–C4; Figures 1R, S1Q, and S1R), previously associated with adaptation to therapy-induced stress.^40,41^ Together, these data support a model in which dual KRAS-EGFR inhibition induces a sequential transition from a diapause-like DTP state to the Paneth-like state in *KRAS*-mutant CRC.

### Paneth-like cells emerge via *trans*-differentiation in response to combined KRAS and EGFR inhibition

**Figure 2.**
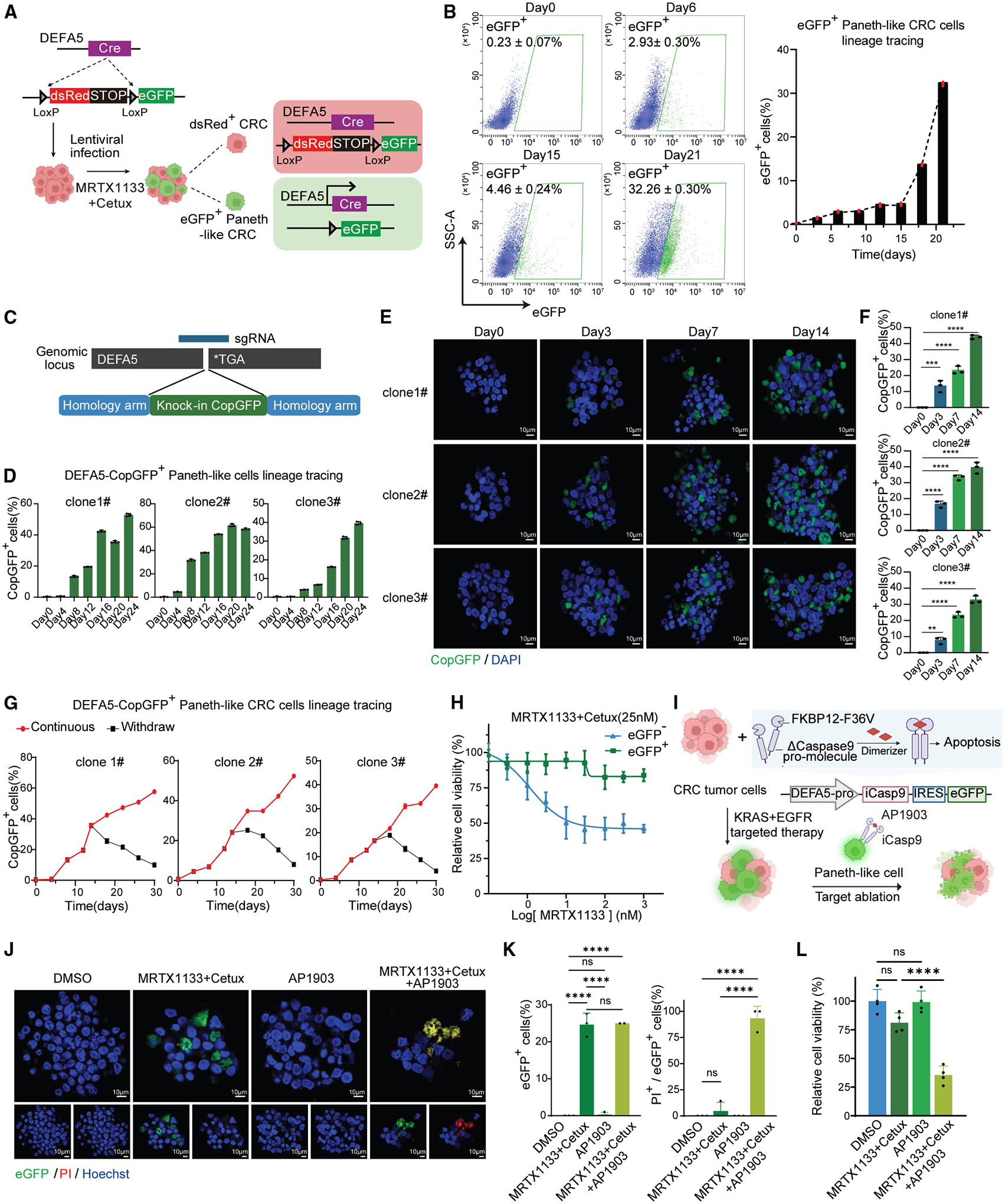
Emergence of Paneth-like cells via *trans*-differentiation in response to combined KRAS and EGFR inhibition (A and B) Schematic illustration showing DEFA5 promoter-driven Cre recombinase and CMV promoter-driven LoxP-dsRed-STOP-Loxp-eGFP system for tracing Paneth-like CRC cells (eGFP^+^) under MRTX1133 + cetuximab treatment (A). Flow analysis of eGFP^+^ Paneth-like cells at indicated time points (*n* = 3 repeats) (B). (C) Lineage tracing of Paneth-like CRC cells using a DEFA5-CopGFP homology-directed repair (HDR) knockin construct. Schematic illustration of the HDR strategy used to insert the CopGFP DNA into the genomic locus before the DEFA5 stop codon using the CRISPR-Cas9 editing system. (D–F) Flow cytometry (D), representative images (E), and quantification (F) of CopGFP^+^ Paneth-like CRC cells (*n* = 3 repeats) over time. Scale bars: 10 μm. (G) Quantification of CopGFP^+^ Paneth-like CRC cells by flow cytometry in representative DEFA5-CopGFP knockin LS174T single clones at different time points during continuous treatment and following withdrawal of MRTX1133 + cetuximab (*n* = 3 repeats). (H) Cell viability of sorted eGFP^−^ and eGFP^+^ Paneth-like CRC cells in LS174T 3D spheroids treated with MRTX1133 + cetuximab (*n* = 6 repeats). (I) Schematic of the DEFA5 promoter-driven inducible caspase-9 and eGFP (DEFA5 pro-iCasp9-IRES-eGFP) construct for labeling and selectively targeting Paneth-like cells. (J and K) Confocal images (J) and quantification of eGFP^+^ cells and PI^+^ cells within the eGFP^+^ population (K) in DEFA5 pro-iCasp9- IRES-eGFP spheroids under various treatment (*n* = 3 fields). Scale bars: 10 μm. (L) Cell viability of DEFA5 pro-iCasp9-IRES-eGFP spheroids after 14 days of drug induction, treated with MRTX1133 (300 nM) + cetuximab (25 nM), with or without AP1903 (100 nM) (*n* = 4 repeats). Data are represented as mean ± SD (B, D, F, G, H, K, and L). Statistical analyses were conducted using one-way ANOVA (F, K, and L). **p < 0.01, ***p < 0.001, ****p < 0.0001. ns, not significant. See also Figure S2.

Paneth cells are typically absent in the colon and considered to originate from LGR5^+^ intestinal stem cells during intestinal homeostasis.^42^ Recent studies find that Paneth-like cell state exists in human CRC.^43,44^ *Lyz*^+^ Paneth-like cells in colorectal adenomas have been reported to be originated from *Lgr5*^+^ intestinal stem cells following genetic ablation of *Apc* in the context of inflammation.^45^ To track the kinetics of Paneth-like state during combined KRAS and EGFR inhibition, we transduced *KRAS*-mutant CRC cells with lentivirus carrying CMV promoter-driven LoxP-dsRed-STOP-LoxP-eGFP and Paneth cell-specific DEFA5 promoter-driven Cre recombinase. In this tracing system, non-Paneth CRC cells expressed dsRed; while in Paneth-like CRC cells, DEFA5 promoter is active and drives Cre expression, which removes dsRed and STOP codon flanked by two LoxP sites and initiates eGFP expression (Figure 2A). We monitored the kinetics of Paneth-like CRC cells following MRTX1133 and cetuximab treatment through fluorescence-activated cell sorting analysis. The eGFP^+^ cells were detected in 0.23% of the pre-treatment LS174T tumor spheroids. Following combined MRTX1133 and cetuximab treatment, the proportion of eGFP^+^ cells gradually increased to 32.26% (Figure 2B), consistent with enrichment of Paneth-like CRC cells in residual tumors. These eGFP^+^ Paneth-like CRC cells showed upregulation of other Paneth cell markers, as confirmed by qPCR (Figure S2A). To further study whether Paneth-like CRC cells arise from the expansion of pre-existing Paneth-like cells or *trans*-differentiation of other CRC cells, we knocked a CopGFP reporter into the endogenous DEFA5 locus (DEFA5-CopGFP) of LS174T cells (Figure 2C). In multiple CopGFP knockin single clones, we consistently observed that CRC cells switched from a CopGFP^−^ non-Paneth to a CopGFP^+^ Paneth-like state in response to KRAS-EGFR inhibition (Figures 2D–2F). To confirm that the shift toward a Paneth-like state in CRC cells is directly induced by KRAS-EGFR inhibition, CRC spheroids were treated with inhibitors for 14 days, followed by either continued drug treatment or drug withdrawal for an additional 16 days. The proportion of CopGFP^+^ Paneth-like CRC cells was tracked over time. While prolonged drug treatment led to a steady increase in the CopGFP^+^ Paneth-like fraction, drug withdrawal resulted in a rapid decline (Figure 2G). These results indicate that the Paneth-like state is directly driven by KRAS-EGFR inhibition and reversible upon drug cessation. To investigate whether the transition to a Paneth-like state confers tumor cells with resistance to the combined therapy, we sorted eGFP^−^ cells and eGFP^+^ Paneth-like cells from CRC spheroids following MRTX1133 plus cetuximab treatment and assessed their sensitivity to dual therapy. We found that the eGFP^+^ Paneth-like CRC cells exhibited a diminished response to dual inhibition of KRAS and EGFR when compared to eGFP^−^ non-Paneth CRC cells (Figure 2H). To functionally assess the role of Paneth-like cells in resistance to dual therapy, we engineered LS174T CRC spheroids to express iCasp9 and eGFP under the control of the DEFA5 promoter, enabling selective ablation of Paneth-like cells upon exposure to the dimerizer AP1903 (Figure 2I).^46^ KRAS-EGFR inhibition led to enrichment of eGFP^+^ Paneth-like cells, which could be efficiently depleted by AP1903. While AP1903 treatment alone had little impact on CRC spheroid growth, its combination with dual KRAS and EGFR inhibition markedly enhanced treatment sensitivity (Figures 2J–2L). Further mechanistic studies demonstrated that eGFP^+^ Paneth-like cells maintained higher basal KRAS-GTP levels, were less responsive to treatment, and exhibited stronger rebound compared to eGFP^−^ cells (Figure S2B). This was associated with elevated pERK levels at both baseline and post treatment (Figure S2C). Similarly, the inhibition of ERK-dependent transcription by the drug was diminished in these cells (Figure S2D). Furthermore, co-staining for DEFA5 and pERK in both iKAP tumors and LS174T xenografts treated with MRTX1133 and cetuximab revealed significantly elevated pERK levels in DEFA5^+^ cells compared to DEFA5^−^ cells, indicating that mitogen-activated protein kinase (MAPK) reactivation occurs preferentially within the Paneth-like cells (Figures S2E and S2F). These data indicate that Paneth-like CRC cells escape KRAS-EGFR inhibition through enhanced MAPK pathway reactivation.

### CRISPR screening identifies SMAD1 as a driver of Paneth-like state transition and therapy resistance

**Figure 3.**
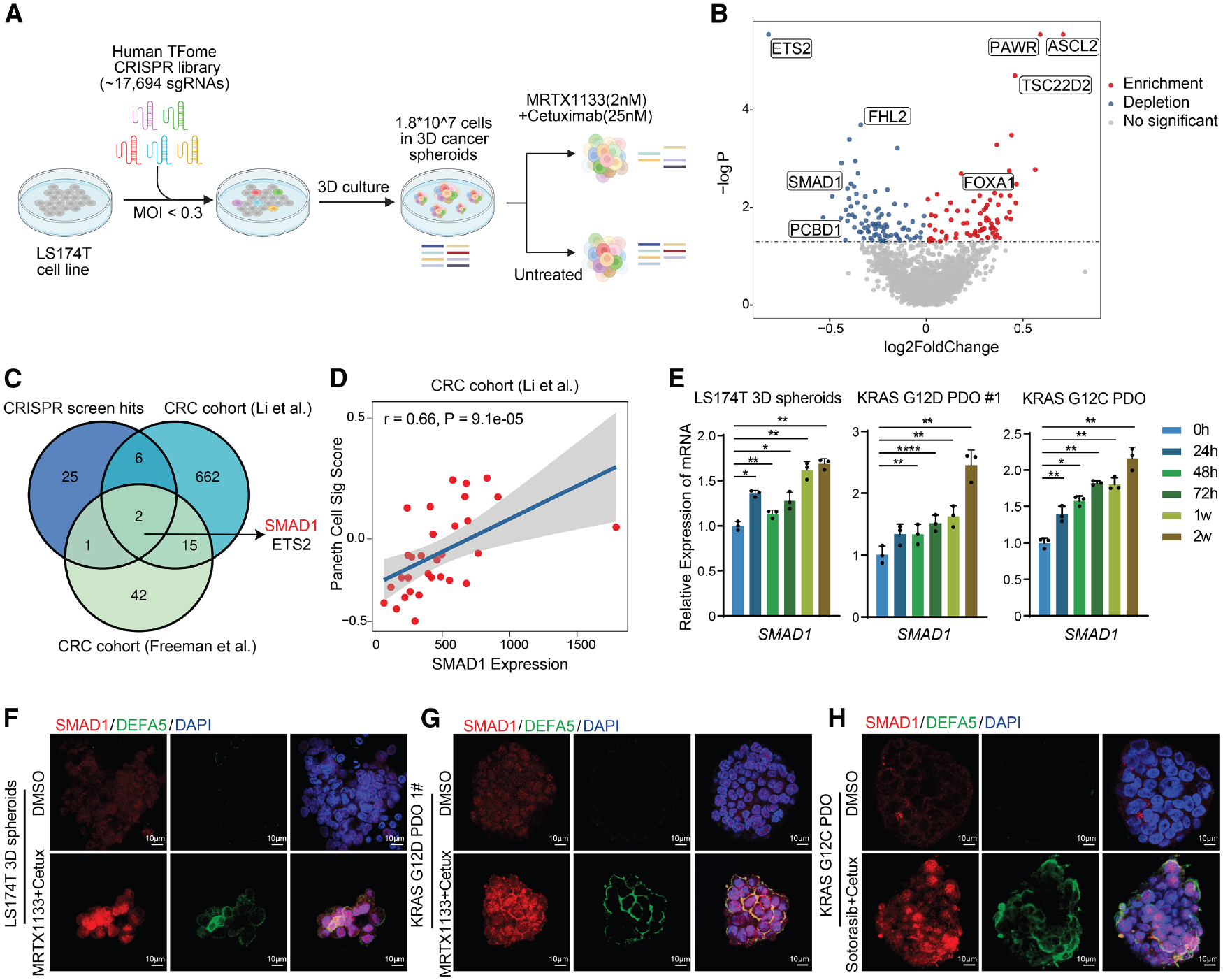
CRISPR screening identifies SMAD1 as a driver of Paneth-like state transition and therapy resistance (A) Schematic of TFome CRISPR screen in LS174T spheroids treated with MRTX1133 + cetuximab. (B) MAGeCK volcano plot showing depleted (blue) and enriched (red) genes from the CRISPR screen. (C) Venn diagram showing the overlap between depletion genes from CRISPR screen and transcription factors positively correlated with Paneth cell signature in public human CRC bulk RNA-seq data.^47,48^ (D) Scatterplots showing the correlation between Paneth cell signature and SMAD1 expression in patients with CRC.^48^ Pearson correlation coefficients and *p* values are shown. (E) RT-qPCR analysis of *SMAD1* expression in LS174T 3D spheroids, KRAS G12D PDO#1, and KRAS G12C PDO upon time-course treatment with KRASi (100 nM) + cetuximab (25 nM). Expression levels were normalized to the reference gene *GAPDH*. Data are presented as mean ± SD (*n* = 3), analyzed by one-way ANOVA, *p < 0.05, **p < 0.01, ****p < 0.0001. (F–H) Representative immunofluorescence images showing co-expression of DEFA5 and SMAD1 in treated spheroids and PDOs. Scale bars: 10 μm. See also Figure S3.

Since the Paneth-like state is associated with marked resistance to KRAS-EGFR inhibition, we aimed to identify transcription factors (TFs) that drive the lineage switch. An ideal candidate TF should be co-expressed with the Paneth-like state and drive resistance to the combined MRTX1133 and cetuximab treatment. To identify such TFs, we conducted an unbiased CRISPR knockout (KO) screen targeting 1,741 human TFs using a library of 17,394 single-guide RNAs (sgRNAs) and 300 negative controls (Figure 3A). After lentiviral-mediated library infection, LS174T cells were cultured as 3D tumor spheroids and treated with either DMSO or 2 nM MRTX1133 combined with 25 nM cetuximab. These drug concentrations were chosen to approximate IC20, enabling identification of TFs whose loss sensitizes cells to the combined treatment (Figure S3A). By comparing sgRNA depletion in the treated group versus the vehicle, we identified 34 TFs whose loss enhanced sensitivity to the combination therapy (Figure 3B). Notably, sgRNAs targeting *ETS2* were significantly depleted, consistent with a previous finding of its emergence as a resistance hit through a gain-of-function screen for resistance to MAPK pathway inhibitors in *BRAF V600E* melanoma.^49^

To further decipher which of these TFs enforce Paneth lineage identity of CRC cells, we analyzed those most strongly associated with the Paneth cell signature in tumors from two published CRC cohorts (Figure 3C, 3D, and S3B).^47,48^ As a major receptor-activated SMADs (R-SMADs) downstream of BMP pathway, SMAD1 has been reported to repress *Lgr5*^+^ stem cell signature genes, thereby limiting stem cell expansion and maintaining intestinal homeostasis.^50–52^ The link between SMAD1 and intestinal development prompted us to investigate its potential role in driving lineage plasticity in CRC.

Combined treatment with MRTX1133 and cetuximab induced an elevation of *SMAD1* transcripts across multiple *KRAS*-mutant CRC tumor spheroids and PDOs (Figures 3E, S3C, and S3D). Using multiplexed immunofluorescence, we demonstrated that combined MRTX1133 and cetuximab treatment significantly increased the proportion of cells expressing SMAD1. Notably, we observed co-localization of SMAD1 with the Paneth cell marker DEFA5 in both LS174T tumor spheroids and two *KRAS G12D* PDOs after treatment (Figures 3F, 3G, and S3E). Similarly, KRAS-EGFR inhibition led to concurrent upregulation of SMAD1 and DEFA5 in two *KRAS G12C* CRC tumor spheroids (SW1463 and RW7213) and a PDO model (Figures 3H, S3F, and S3G). The upregulation of SMAD1 in therapy-surviving Paneth-like CRC cells, along with its identification as a drug sensitization hit in our CRISPR dropout screen, suggests that SMAD1 might play a role in driving resistance to KRAS-EGFR combination therapy through promoting the transition to a Paneth-like cell state.

### SMAD1 drives resistance to KRAS-EGFR combination therapy by promoting Paneth-like state transition

**Figure 4.**
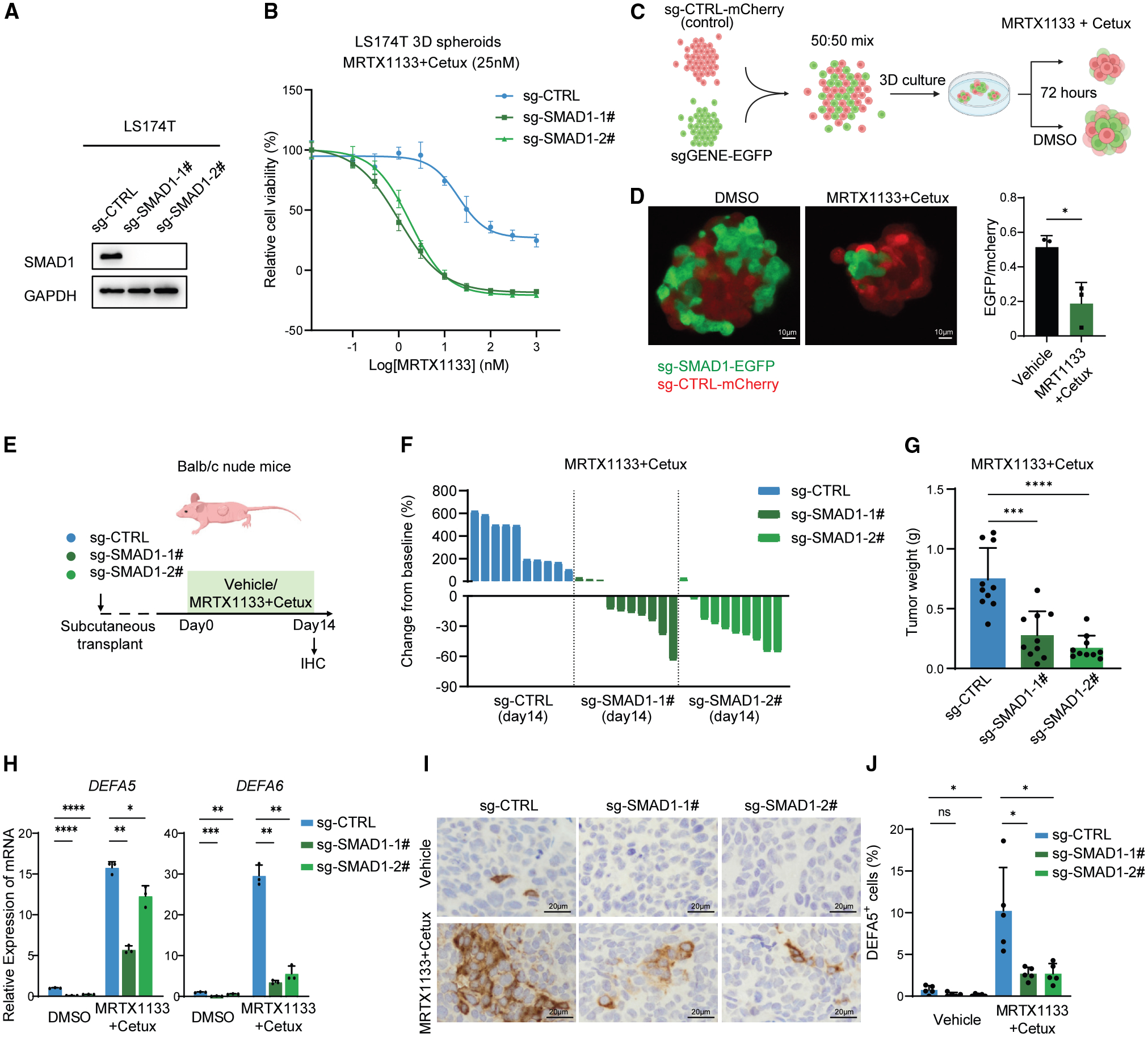
SMAD1 drives resistance to KRAS-EGFR combination therapy by promoting Paneth-like state transition (A) Western blot of SMAD1 in LS174T sg-CTRL cells (sg-CTRL) and SMAD1 KO clones (sg-SMAD1–1# and 2#). GAPDH was used as a loading control. (B) Cell viability of LS174T control and SMAD1 KO cells treated with increasing concentrations of MRTX1133 + cetuximab (25 nM) (*n* = 6). (C) Schematic for the competitive growth assay in mCherry-labeled sg-CTRL LS174T cells and EGFP-labeled sg-SMAD1 LS174T cells. (D) Fluorescence microscopy images (left) showing mCherry and EGFP in LS174T 3D spheroids treated with either DMSO or MRTX1133 (2nM) + cetuximab (25 nM). The bar graph (right) quantifies EGFP:mCherry ratios of DMSO and MRTX1133 + cetuximab-treated groups (*n* = 3 fields). Scale bars: 10 μm. (E–G) LS174T control and SMAD1 KO cells were subcutaneously implanted into BALB/c nude mice (E). Tumor volume changes (F) and tumor weights (G) were measured following 2 weeks of MRTX1133 + cetuximab treatment (*n* = 10 per group). (H) RT-qPCR analysis of *DEFA5* and *DEFA6* transcripts in LS174T control and SMAD1 KO cells treated with DMSO or MRTX1133 + cetuximab for 72 h (*n* = 3). Expression levels were normalized to the reference gene *GAPDH*. (I and J) Immunohistochemical staining of DEFA5 in xenograft tumors derived from LS174T control and SMAD1 KO cells treated with vehicle or MRTX1133 + cetuximab (I), with corresponding quantification (*n* = 5 tumors) (J). Scale bars: 20 μm. Data are represented as mean ± SD (B, D, G, H, and J). Statistical analyses were conducted using two-tailed *t* test (D) or one-way ANOVA (G) or Bonferroni-corrected multiple *t* tests (H and J). *p < 0.05, **p < 0.01, ***p < 0.001, ****p < 0.0001, ns, not significant. See also Figures S4 and S5; Table S1.

To investigate the role of SMAD1 in resistance to combined KRAS and EGFR inhibition, we generated *SMAD1*-KO LS174T and LS180 cells and assessed their sensitivity to MRTX1133 and cetuximab treatment. *SMAD1* KO significantly increased the sensitivity of CRC tumor spheroids to this combination therapy (Figures 4A, 4B, and S4A–S4C). Competitive growth assays using LS174T spheroids further validated this increased susceptibility. Specifically, LS174T cells were transduced with lentivirus expressing either mCherry along with a non-targeting control sgRNA, or EGFP along with *SMAD1*-targeting sgRNA and then pooled at a 1:1 mCherry:EGFP ratio (Figure 4C). While *SMAD1* KO had minimal effect on cell survival under vehicle treatment, the proportion of EGFP^+^ sgSMAD1^+^ cells in the chimeric spheroids markedly decreased upon MRTX1133 and cetuximab treatment, indicating that *SMAD1* loss rendered cells highly susceptible to KRAS-EGFR inhibition (Figure 4D). We next assessed the *in vivo* impact of *SMAD1* KO using the LS174T subcutaneous xenograft model (Figure 4E). *SMAD1* depletion did not affect baseline tumor growth. However, its loss significantly reduced pERK and restored tumor sensitivity to MRTX1133 and cetuximab, inducing regression in 70%–90% of mice (Figures 4F, 4G, S4D, and S4E). Transcriptomic profiling of *SMAD1*-KO cells showed attenuated Paneth signature enrichment upon KRAS-EGFR inhibition (Figure S4F), corroborated by reduced Paneth marker induction by qPCR analysis (Figure 4H). Moreover, immunostaining of DEFA5 in LS174T xenograft tumors demonstrated that *SMAD1* KO largely abrogated the Paneth-like state transition induced by KRAS-EGFR inhibition, highlighting SMAD1’s critical role in promoting Paneth cell differentiation programs (Figures 4I and 4J). Additionally, *SMAD1* overexpression in LS174T spheroids upregulated *DEFA5* and *DEFA6* and conferred resistance to dual therapy (Figures S4G–S4I).

We next assessed whether BMP signaling is involved in therapy-induced SMAD1 activation. Unexpectedly, we found that canonical BMP targets *ID1* and *ID3* were downregulated following dual KRAS-EGFR inhibition (Figure S5A). Moreover, exogenous BMP2 or BMP4 stimulation failed to induce Paneth markers or alter sensitivity to dual therapy (Figures S5B–S5E), indicating that SMAD1 is activated via a non-canonical route during therapy-induced lineage plasticity. Consistently, other R-SMADs in the canonical BMP pathway, including SMAD5 and SMAD9, were dispensable for Paneth-like transition and resistance (Figures S5F–S5L). Moreover, Paneth-like cells were readily induced by KRAS-EGFR inhibition in both *SMAD4* wild-type models (LS174T and KRAS G12D PDOs) (Figures 1I–1N, S1J, and S1K) and *SMAD4* mutant lines (SW1463 and RW7213) harboring the loss-of-function *SMAD4 R361C* mutation (Figures S1L–S1O).^53^ Analysis of The Cancer Genome Atlas (TCGA) datasets revealed no significant difference in Paneth-like gene signature expression between *SMAD4* wild-type and mutant CRCs (Figure S5M).^54^ In addition, we detected no *SMAD4* genetic alterations or expression change in response to dual KRAS-EGFR inhibition across patient tumors, PDXs, and CRC spheroids (Table S1; Figure S5N). Functionally, *SMAD4* knockdown did not impair therapy-induced Paneth-like transition or affect treatment sensitivity (Figures S5O–S5Q). This aligns with prior findings that *SMAD4* deletion does not alter Paneth cell numbers during intestinal development in mice, suggesting that SMAD4 is not required for Paneth cell specification.^55^ Together, these findings establish SMAD1 as a non-redundant driver of Paneth-like plasticity and therapeutic resistance that acts independently of SMAD4 and canonical BMP signaling.

### SMAD1-FGFR3 signaling axis drives Paneth-like state transition and promotes MAPK reactivation in Paneth-like cells

**Figure 5.**
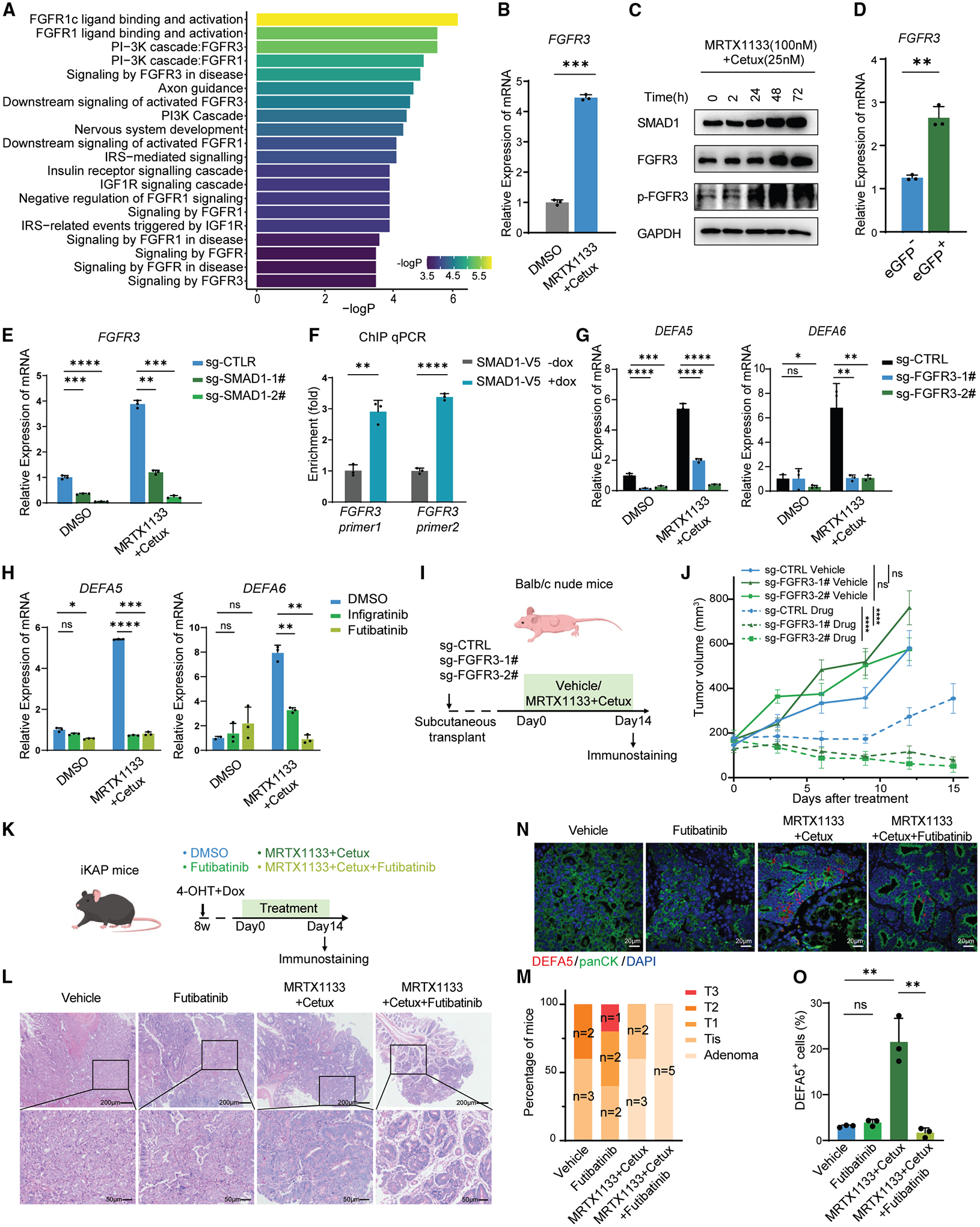
SMAD1-FGFR3 signaling axis drives Paneth-like state transition and promotes MAPK reactivation in Paneth-like cells (A) Bar plot depicting the results of pathway enrichment analysis for the top 500 genes positively correlated with SMAD1, based on the REACTOME pathway database. (B) RT-qPCR analysis of *FGFR3* mRNA expression in LS174T 3D spheroids treated for 72 h (*n* = 3). Expression levels were normalized to the reference gene *GAPDH*. (C) Western blot analysis of SMAD1, FGFR3, and p-FGFR3 expression in LS174T 3D spheroids treated with MRTX1133 (100 nM) + cetuximab (25 nM) over time. GAPDH was used as a loading control. (D) RT-qPCR analysis of *FGFR3* mRNA expression in sorted eGFP^+^ Paneth-like CRC cells compared with eGFP^−^ cells (*n* = 3). Expression levels were normalized to the reference gene *GAPDH*. (E) RT-qPCR analysis of *FGFR3* mRNA expression in LS174T control and SMAD1 KO cells with or without MRTX1133 (100 nM) + cetuximab (25 nM) (*n* = 3). Expression levels were normalized to the reference gene *GAPDH*. (F) ChIP-qPCR showing the increased binding of SMAD1 to the FGFR3 promoter upon doxycycline-induced SMAD1-V5 tag overexpression in LS174T cells (*n* = 3). (G) RT-qPCR analysis of *DEFA5* and *DEFA6* mRNA expression in LS174T control and FGFR3 KO cells with or without MRTX1133 (100 nM) + cetuximab (25 nM) (*n* = 3). Expression levels were normalized to the reference gene *GAPDH*. (H) RT-qPCR analysis of *DEFA5* and *DEFA6* mRNA expression in LS174T 3D spheroids following treatment with MRTX1133 + cetuximab, with or without FGFR inhibitors infigratinib (300 nM) and futibatinib (300 nM) (*n* = 3). Expression levels were normalized to the reference gene *GAPDH*. (I) Schematic of *FGFR3* KO LS174T xenograft experiments. (J) Tumor growth curves comparing LS174T control (sg-CTRL) and FGFR3 KO (sg-FGFR3–1#, sg-FGFR3–2#) xenografts over time with or without MRTX1133 + cetuximab treatment (*n* = 5 per group). (K) Schematic of iKAP mice treatment experiments. iKAP mice with 80% tumor occlusion of the lumen were randomized into different treatment groups: vehicle, futibatinib (15 mg/kg, oral, once daily), MRTX1133 (30 mg/kg, intraperitoneal, twice daily) + cetuximab (50 mg/kg, intraperitoneal, twice weekly), and MRTX1133 + cetuximab + futibatinib (*n* = 5 per group). (L and M) Representative histological images (L) and quantification of the percentage of T classifications (M) in iKAP tumors across treatment groups. Scale bars: 200 μm (overview, top) and 50 μm (magnified, bottom). (N and O) Immunofluorescence staining of DEFA5, panCK, and DAPI in iKAP tumors (N), with corresponding quantification (*n* = 3 tumors) (O). Scale bars: 20 μm. All RT-qPCR and ChIP-qPCR data represent mean ± SD of 3 independent replicates (B, D, E, F, G, and H). Data are represented as mean ± SEM (J) or mean ± SD (O). Statistical analyses were conducted using two-tailed *t* test (B, D, and F), Bonferroni-corrected multiple *t* tests (E, G, and H), two-way ANOVA (J), or one-way ANOVA (O). *p < 0.05, **p < 0.01, ***p < 0.001, ****p < 0.0001, ns, not significant difference. See also Figures S6 and S7.

To delineate SMAD1’s role in driving Paneth-like lineage plasticity, we conducted pathway enrichment analysis on the top 500 genes most strongly correlated with *SMAD1* expression using RNA-seq data from LS174T sg-CTRL and sg-SMAD1 cells treated with vehicle or MRTX1133 and cetuximab. Among the enriched pathways, multiple FGFR1 and FGFR3 signaling-related pathways were identified (Figure 5A). Consistent with this, analysis of primary CRC samples from previously published cohorts^49^ revealed strong positive correlations between *SMAD1* expression, FGFR1 and FGFR3 signaling activity, and Paneth cell signatures (Figures S6A and S6B). We assessed *FGFR1* and *FGFR3* expression via qPCR and found *FGFR1* barely detectable in LS174T tumor spheroids and two PDOs. In contrast, *FGFR3* was readily expressed in these models and further upregulated upon combined KRAS and EGFR inhibition (Figures 5B and S6C). Western blot revealed increases in both total and phosphorylated FGFR3 levels, along with elevated SMAD1 expression post treatment in LS174T cells (Figure 5C). We then assessed the expression of known FGFR3 ligands^56,57^ and found concomitant enrichment of *FGFR3* and its ligands *FGF20* and *FGF23* in eGFP^+^ Paneth-like cells, accompanied by elevated p-FGFR3 levels (Figures 5D, S6D, and S6E). Moreover, the transcripts of *FGF20* and *FGF23* were also upregulated by MRTX1133 and cetuximab treatment, while *SMAD1* depletion abolished the upregulation of *FGFR3*, *FGF20*, and *FGF23* (Figures 5E and S6F). Chromatin immunoprecipitation (ChIP)-qPCR analysis further demonstrated that SMAD1 directly binds to the *FGFR3* promoter (Figure 5F). Thus, our data suggest that the FGFR3 pathway is activated via SMAD1-mediated transcriptional regulation in Paneth-like cells.

Previous studies using GEMMs and colon carcinoma-derived epithelial cell lines implicated FGFR3 signaling in Paneth cell lineage specification during intestinal development.^58,59^ To determine whether FGFR3 contributes to the Paneth-like state transition triggered by KRAS and EGFR inhibition, we knocked out *FGFR3* in LS174T spheroids (Figures S6G and S6H). Genetic depletion of *FGFR3* significantly attenuated the upregulation of Paneth cell marker genes induced by KRAS and EGFR inhibition (Figure 5G). We further explored the functional role of FGFR3 using futibatinib and infigratinib, two pan-FGFR inhibitors approved for intrahepatic cholangiocarcinoma with FGFR alterations.^60,61^ While FGFR inhibitors alone had minimal effect on Paneth marker expression, their combination with MRTX1133 and cetuximab suppressed the upregulation of these markers (Figure 5H). Conversely, FGF20 and FGF23 stimulation modestly activated FGFR3 and induced Paneth cell markers, while further amplifying FGFR3 signaling by engaging dual therapy-upregulated FGFR3. This led to synergistic upregulation of both *DEFA5* and *DEFA6* upon co-treatment with MRTX1133 and cetuximab (Figures S6I and S6J). More importantly, both *FGFR3* KO and FGFR inhibitor treatment re-sensitized LS174T colospheres to combined KRAS and EGFR inhibition, underscoring the causative role of FGFR3 in Paneth-like transition and therapy resistance (Figures S7A–S7D). We next sought to determine whether FGFR3 drives resistance by reactivating MAPK signaling specifically in Paneth-like cells. To this end, we performed *FGFR3* knockdown and its pharmacological inhibition in sorted eGFP^+^ Paneth-like and eGFP^−^ CRC cells. These interventions suppressed FGFR3 activation, attenuated MAPK rebound, and restored the drug sensitivity of eGFP^+^ cells to levels comparable to eGFP^−^ counterparts (Figures S7E–S7I). Consistent with *in vitro* findings, *FGFR3* KO enhanced ERK suppression and markedly sensitized LS174T xenograft tumors to dual therapy, resulting in tumor regression in all treated mice (Figures 5I, 5J, and S7J). Moreover, futibatinib co-treatment with MRTX1133 and cetuximab more robustly suppressed MAPK signaling and significantly impaired the progression of *KRAS G12D*-mutant iKAP tumors, leading to complete reversion of malignant CRC cells to benign adenoma (Figures 5K–5M and S7K–S7N). Notably, futibatinib monotherapy had no obvious effect on iKAP tumor growth (Figures 5M and S7M), highlighting the specific role of FGFR3 in mediating lineage plasticity and resistance to KRAS and EGFR dual inhibition. The reversal of lineage plasticity was further evidenced by DEFA5 immunostaining, which demonstrated that MRTX1133 and cetuximab treatment-induced DEFA5 upregulation was significantly attenuated upon co-treatment with futibatinib (Figures 5N–5O). Together, these findings demonstrate that dual KRAS-EGFR inhibition induces a SMAD1-dependent upregulation of FGFR3 and its ligands FGF20 and FGF23, which activates FGFR3 signaling. FGFR3 promotes both Paneth-like plasticity and MAPK reactivation, driving resistance to dual KRAS-EGFR inhibition.

### Paneth-like state is enriched in human residual CRC tumors following combined KRAS-EGFR inhibition

**Figure 6.**
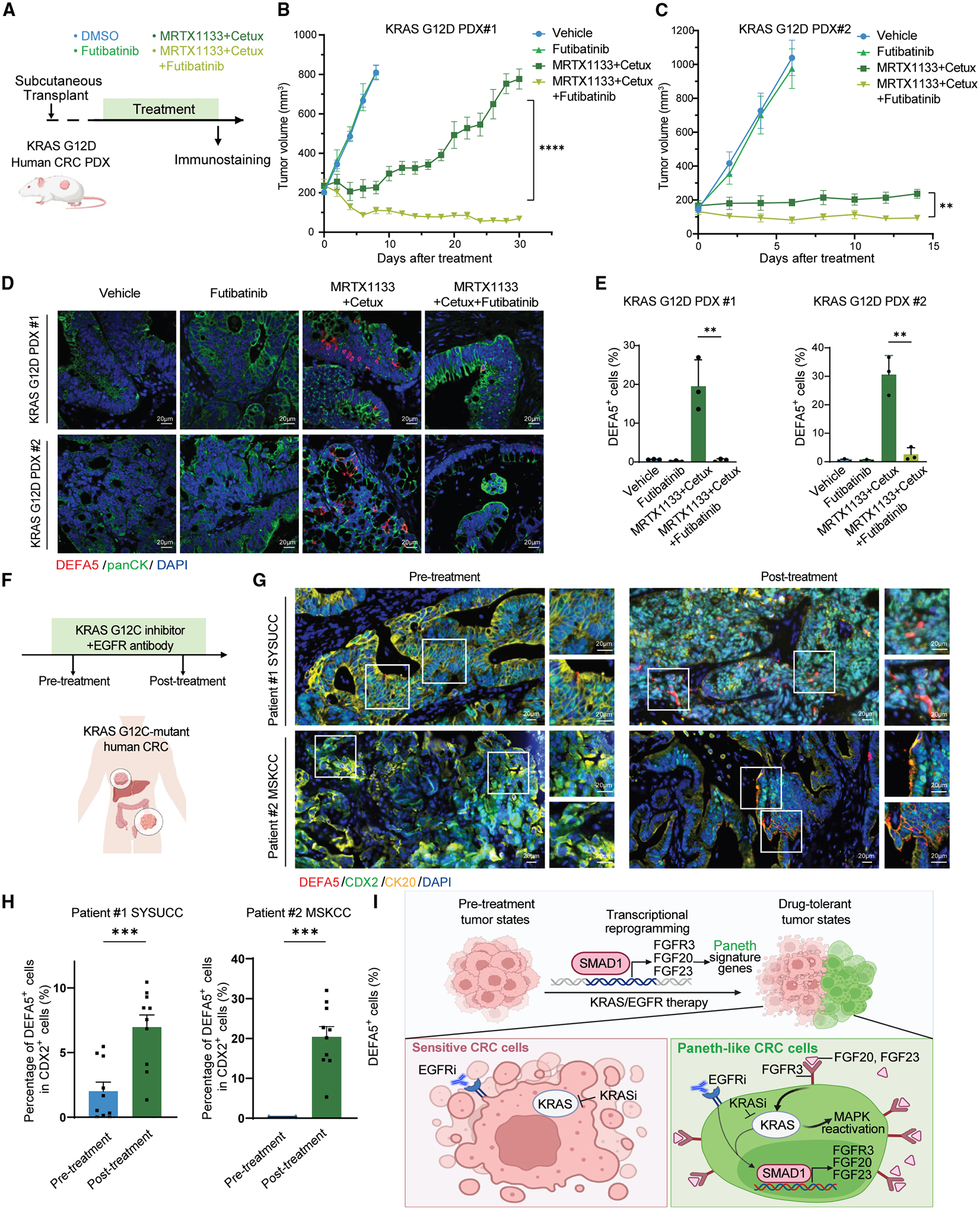
Paneth-like state enrichment in human residual CRC tumors following combined KRAS-EGFR inhibition (A) Schematic illustration of experimental design in human KRAS G12D CRC PDX models. (B and C) Tumor growth curves of KRAS G12D PDX#1 (B) and PDX#2 (C) tumors treated with vehicle, futibatinib (15 mg/kg, oral, once daily), MRTX1133 (30 mg/kg, intraperitoneal, twice daily) + cetuximab (50 mg/kg, intraperitoneal, twice weekly), or MRTX1133 + cetuximab + futibatinib (*n* = 5–6 per group). (D and E) Immunofluorescence staining of DEFA5, PanCK, and DAPI in each treatment group (D), with corresponding quantification analysis (*n* = 3 tumors) (E). Scale bars: 20 μm. (F) Schematic representation of tumor tissue biopsies obtained from two patients with *KRAS G12C*-mutant CRC before and after KRAS-EGFR-targeted therapy. (G and H) Immunofluorescence staining of DEFA5, CDX2, CK20, and DAPI in pre-treatment and post-treatment tumor biopsies (G), with corresponding quantification analysis from 10 independent fields of view (H). Scale bars: 20 μm. (I) Schematic summary of key findings. Data are represented as mean ± SEM (B and C) or mean ± SD (E and H). Statistical analyses were conducted using two-way ANOVA (B and C), or two-tailed *t* test (E and H). **p < 0.01, ***p < 0.001, ****p < 0.0001. See also Figure S8; Table S2.

To determine whether FGFR3 confers resistance to combined KRAS and EGFR inhibition in human CRC, we generated two PDX models from patients with *KRAS G12D*-mutant CRC and treated tumor-bearing mice with vehicle, KRAS inhibitor and cetuximab, futibatinib, or the triple combination. Dual KRAS and EGFR inhibition initially stabilized disease, followed by disease progression in one model. Futibatinib treatment alone showed no efficacy. However, triple inhibition of KRAS, EGFR, and FGFR3 led to marked suppression of p-FGFR3 and induced profound and sustained tumor regression without significant body weight loss (Figures 6A–6C and S8A–S8G). Consistent with findings in iKAP mouse models, the combination of KRAS inhibitor and cetuximab induced upregulation of Paneth cell marker DEFA5 in both PDX tumors, an effect reversed by futibatinib co-treatment (Figures 6D and 6E). These results highlight FGFR3 as a promising therapeutic target to counteract lineage plasticity-driven resistance in *KRAS*-mutant CRC.

To validate clinical relevance, we analyzed paired biopsies from two patients with *KRAS G12C*-mutant CRC who received KRAS G12C inhibitors in combination with EGFR antibodies (garsorasib + cetuximab for patient 1, sotorasib + panitumumab for patient 2) (Figure 6F; Table S2). In patient 1, a primary tumor biopsy was collected prior to treatment, and a liver metastasis biopsy was obtained after 13 months of treatment; in patient 2, a liver biopsy was taken just before treatment, and a residual peritoneal lesion was removed after 8 months of treatment. We found that Paneth-like cells were markedly enriched following combination therapy, as evidenced by DEFA5 immunostaining in tumors from both patients (Figures 6G and 6H). Taken together, these findings establish Paneth-like plasticity as a conserved resistance mechanism across murine and human *KRAS*-mutant CRC models. Mechanistically, SMAD1 activates FGFR3 to drive both Paneth-like cell transition and MAPK reactivation, thereby sustaining MAPK pathway activity in this therapy-refractory cell population. Co-targeting FGFR3 with KRAS-EGFR inhibition offers a compelling strategy to mitigate lineage plasticity-driven resistance and improve therapeutic outcomes (Figure 6I).

## DISCUSSION

Lineage plasticity has emerged as a critical mechanism of non-genetic resistance to KRAS-targeted therapies in NSCLC and PDAC.^27–31^ However, its role in mediating resistance to dual KRAS and EGFR-targeted therapies in CRC remains unexplored. In this study, we identify a Paneth-like cell state that contributes to non-genetic resistance to KRAS-EGFR inhibition in CRC. We observed enrichment of the Paneth-like cell state in *KRAS*-mutant GEMMs, PDOs, xenografts, and biopsies from patients with CRC treated with KRAS and EGFR dual therapy. Using genetic reporter systems to track cell states over time, we found that CRC cells evade this therapy by acquiring lineage plasticity, transitioning into a Paneth-like cell state. scRNA-seq analysis revealed that CRC cells transit through a diapause-like DTP state prior to acquiring a Paneth-like phenotype. Similar intermediate states have been described during embryonic development, where cells enter a paused, pluripotent state in response to environmental stress.^62^ In cancer, such diapause-like states enable survival under therapeutic pressure and eventually give rise to drug-resistant populations.^40,41^ The Paneth-like state exhibited features similar to residual CRC cells surviving EGFR antibody treatment alone in PDX models,^25^ suggesting that the transition to a Paneth-like state could be a broader adaptive mechanism to therapies targeting the RTK-RAS pathway in CRC. The loss of Paneth-like identity upon drug withdrawal further corroborates that this state is directly driven by KRAS-EGFR inhibition. These findings emphasize the importance of identifying convergent cell states that tolerate RTK-RAS pathway inhibitors, presenting opportunities to target the dependencies of Paneth-like CRC cells or block key mediators of lineage plasticity.

We found that this adaptive strategy varies across cancer types. In NSCLC, *KRAS*-mutant tumors evade therapy through transition into AT1-like,^29^ squamous,^27,28^ or mucinous states,^29^ while pancreatic cancer cells adopt a classical epithelial state to bypass KRAS inhibition.^30,31^ Our findings suggest that CRC cells switch to a Paneth-like state as their survival strategy. These differences likely reflect lineage-specific features of the tissue of origin, shaping divergent resistance trajectories across different tumor types. Understanding these distinct paths is crucial for designing treatments tailored to each cancer type. Future studies integrating single-cell transcriptomic profiling with lineage tracing will be essential to dissect tissue-specific resistance trajectories and uncover lineage-dependent vulnerabilities.

Mechanistically, we identified SMAD1 as a key driver of this Paneth-like transition and resistance. Although SMAD1 has been shown to regulate intestinal homeostasis by constraining *Lgr5*^+^ stem cell expansion,^52^ its role in treatment-induced lineage plasticity remains under-explored. Here, we show that SMAD1 increases after KRAS-EGFR inhibition, triggering the Paneth-like transition. While SMAD1 is typically activated through BMP receptor-mediated phosphorylation,^63^ we found that SMAD1 transcripts are increased after KRAS-EGFR combined treatment, suggesting that alternative transcriptional or epigenetic mechanisms contribute to its upregulation. We further show that SMAD1 activates FGFR3 through upregulation of the expression of both FGFR3 and FGF ligands, which triggers an FGFR3-dependent Paneth-like transition. Silencing SMAD1 prevented this shift and restored sensitivity to combination treatment, highlighting its essential role in lineage plasticity and therapy resistance. Genetic analysis and functional studies further establish SMAD1 as a non-redundant driver of Paneth-like plasticity and therapeutic resistance that functions independently of SMAD4 and canonical BMP pathway.

We show that the Paneth-like state fuels resistance by maintaining active MAPK signaling. Compared to non-Paneth CRC cells in treated tumors, Paneth-like cells showed stronger baseline MAPK activity and weaker response to KRAS-EGFR inhibition, as evidenced by less effective MAPK suppression. This sustained MAPK activity is attributed to FGFR3 activation, in line with its role in conferring RAF inhibitor resistance in *BRAF*-mutant melanoma.^64^ In our models, FGFR3 sustains MAPK signaling in Paneth-like CRC cells, promoting a resistant phenotype that blunts the efficacy of KRAS-EGFR combination therapy. Genetic or pharmacological FGFR3 inhibition not only prevents this Paneth-like state enrichment but also restores sensitivity to dual therapy *in vitro* and *in vivo*, underscoring FGFR3 as a driver of plasticity-driven resistance. This echoes FGFR3’s role in Paneth cell lineage specification during intestinal development, where it regulates lineage-specific genes via TCF4 and β-catenin-dependent and -independent pathways.^58,59^ Exploring these downstream signals of FGFR3 could deepen our understanding of lineage plasticity-driven resistance in CRC.

Despite the established role of lineage plasticity in resistance to KRAS-targeted therapies,^26–31^ effective therapeutic interventions targeting drivers of lineage plasticity remain lacking. Our study shows that SMAD1-mediated FGFR3 activation underlies the Paneth-like transition and drug resistance in CRC cells. Genetic or pharmacological inhibition of FGFR3 resensitized *KRAS-*mutant CRC cells to combined KRAS-EGFR inhibition. Several pan-FGFR inhibitors, such as futibatinib and infigratinib, are approved for treating FGFR-altered cancers.^60,61^ Additionally, isoform-selective FGFR3 inhibitors (e.g., LOXO-435) are under clinical investigation.^65^ FGFR inhibitor monotherapy has shown limited efficacy in patients with CRC with FGFR alterations (NCT04096417). Consistent with this, we found that FGFR inhibitor monotherapy had no effect on tumor growth in iKAP mouse models and human CRC PDX models. Strikingly, combining FGFR inhibitors with KRAS-EGFR dual-targeted therapy elicited a robust synergistic antitumor effect. These findings advocate for clinical trials exploring FGFR3 inhibition to overcome plasticity-driven resistance in patients with *KRAS*-mutant CRC.

In summary, our study uncovers a Paneth-like cell transition as a resistance strategy against dual KRAS and EGFR inhibition in CRC. We demonstrate that SMAD1 orchestrates this shift by activating FGFR3, enabling cancer cells to withstand treatment. Targeting the SMAD1-FGFR3 pathway offers a promising approach to overcome therapy resistance, potentially improving outcomes for patients with *KRAS*-mutant CRC (Figure 6I).

### Limitations of the study

Given the limited availability of paired pre- and on-treatment samples, our clinical validation was restricted to two patients with CRC. Despite the small cohort, residual tumors in both cases consistently showed enrichment of Paneth cell markers. Future studies with larger patient cohorts will be required to validate the clinical relevance of Paneth-like transition. Mechanistically, although we identified SMAD1-FGFR3 signaling as a driver of Paneth-like transition and MAPK reactivation, how KRAS-EGFR inhibition induces SMAD1 expression and how SMAD1 regulates FGFR3 remain unresolved. Further mechanistic studies will be needed to define these upstream regulatory events.

## STAR★METHODS

### EXPERIMENTAL MODEL AND STUDY PARTICIPANT DETAILS

#### Mouse models

iKAP CRC mouse model (*tet-O-LSL-Kras*^*G12D*^, *Apc*^*L/L*^*, Trp53*^*L/L*^, *Villin*^*CreERT2*^) was described previously.^32^ NSG mice were obtained from the Guangdong Medical Laboratory Animal Center (Guangzhou, China), and BALB/c nude mice were purchased from Guangdong GemPharmatech Co., Ltd. (Guangzhou, China). Mice were maintained in a specific pathogen-free (SPF) facility under controlled conditions, including a 12/12-hour light/dark cycle, an ambient temperature of 25°C, and 60% relative humidity. All animals had free access to a standard diet and water. Experiments were initiated when the mice reached 6–8 weeks of age. Their overall health status was assessed through routine monitoring. Animal welfare was evaluated daily, and humane endpoints were strictly followed. To minimize physiological variability, only male mice were used for tumor xenograft studies, while both sexes were utilized in the iKAP CRC model. All animal experiments were conducted in compliance with ethical guidelines and approved by the Animal Experimentation Ethics Committee of Sun Yat-sen University Cancer Center (L025504202211004).

#### Cell lines

The colorectal cancer cell lines LS174T and LS180 were obtained from ATCC, RW7213 and SW1463 cells were kindly provided by Dr. Sandra Misale (Johns Hopkins School of Medicine). The LS174T and RW7213 cells were cultured in RPMI 1640 medium (Gibco), LS180 cells were cultured in DMEM medium (Gibco), and SW1463 cells were cultured in Leibovitz’s L15 medium (eLGbio). All the cell lines were cultured at 37°C under 5% CO2 in culture medium containing 10% FBS and 1% penicillin-streptomycin, and regularly tested to ensure the absence of mycoplasma contamination. For 3D spheroid cultures, tissue culture flasks or plates were coated with poly-HEMA overnight (20mg/ml in 95% ethanol; Sigma, Cat# P3932) to prevent cell attachment. Cells were then mixed with growth medium containing 0.75% methylcellulose (Sigma, Cat# M0512) and seeded into poly-HEMA-coated plates for spheroid formation, as described previously.^66^

#### CRC organoids

Colorectal tumors from the *KRAS-G12D* mutant PDX models and a *KRAS-G12C* mutant colorectal cancer patient were dissected into small pieces (1 mm^3^) and incubated in the digestion solution containing 0.125 mg/mL type II collagenase, 0.125 mg/mL type II dispase, 1 μM RHOK inhibitor Y-27632, and 2% fetal bovine serum. The tissue was digested on a rotating shaker at 37°C for 15–30 minutes. After red blood cell removal, tumor cells were harvested by centrifugation at 1200 rpm for 3 minutes. The cell pellet was resuspended in organoid culture medium, mixed with an equal volume of Matrigel, and plated into 24-well plates. The composition of colorectal cancer organoid culture medium was as follows: DMEM/F12 supplemented with 2 mM Glutamax, 10 mM HEPES, 10 mM Nicotinamide, 1 mM N-acetylcysteine, N2, B27, 10 nM Gastrin, 500 nM A83–01, 10 ng/mL FGF2, 50 ng/mL Noggin, 10 μM Y-27632, 10 μM SB202190, and 100 μg/mL primocin. Medium was refreshed every 2–3 days depending on organoid growth. For passaging, organoids were collected, washed with PBS, and incubated with TrypLE (GIBCO, Cat# 12605010) at 37°C for 1–2 minutes to dissociate organoids from Matrigel. Digestion was then stopped by suspending the organoids in PBS containing 1% BSA. The organoid suspension was gently sheared by pipetting, followed by washing with PBS and centrifugation. Finally, the organoids were resuspended in Matrigel and re-seeded at a 1:3 to 1:5 ratio.

#### Human specimens

Paraffin-embedded human tissue samples used in this study were obtained from Sun Yat-sen University Cancer Center (SYSUCC) and Memorial Sloan Kettering Cancer Center (MSK). The research protocol adhered to the principles of the Declaration of Helsinki and was approved by the Ethics Committee of SYSUCC (No. B2024-453-01) and the Institutional Review Board (IRB) of MSK (No. 12–245). Following CRC diagnosis confirmation by experienced pathologists, written informed consent was obtained from all patients. Clinical characteristics, including age, sex, race, tumor stage, and treatment duration, are provided in Table S2. All analyses of MSK samples were performed at MSK.

### METHOD DETAILS

#### Animal experiments

For the iKAP CRC mouse model, colorectal tumors were induced by administering 4-Hydroxytamoxifen (4-OHT) (Sigma Aldrich, Cat# H7904) via enema at 8 weeks of age, followed by providing doxycycline (Macklin, Cat# 10592-13-9) containing drinking water (2g/L) as previously described.^32^ Tumor progression was monitored weekly under colonoscopy using the Storz veterinary endoscope. Mice with ≥80% luminal occlusion by tumor were randomly divided into different groups for further treatment. Upon reaching the endpoint, colons were excised to assess tumor burden by quantifying tumor number and size using a stereo microscope.

For xenograft experiments, 5×10^5^ LS174T cells, LS174T sg-CTRL or sg-FGFR3–1# and 2# cells resuspended in PBS, were mixed with Matrigel (Corning, Cat# 354234) and subcutaneously injected into the right flanks of the BALB/c nude mice. For the patient-derived xenograft (PDX) models, tumor tissues from KRAS G12D CRC patients were dissected into fragments and implanted subcutaneously into the right flank of NSG mice. Tumor size and mouse body weight were routinely monitored every 2 days by a caliper. Tumor volume was monitored and calculated using the formula: (length×width^2^) /2. When the tumor reached around 150–200 mm^3^ in size, the mice were grouped randomly as indicated in each figure. At the endpoint, mice were euthanized, and the tumors were dissected and weighed.

Treatment regimens included MRTX1133 (30 mg/kg, twice daily, intraperitoneally; TargetMol, Cat# T9303), Cetuximab (50 mg/kg, twice weekly, intraperitoneally; Merck, Cat# 10736), Futibatinib (15 mg/kg, once daily, orally; TargetMol, Cat# T5044), administered either as monotherapies or in combinations, as indicated.

#### *In vitro* tumor spheroid and organoid proliferation assays

For tumor spheroids, 3,000 LS174T or LS180 cells were seeded into 96-well plates pre-coated with poly-HEMA in growth medium supplemented with 0.75% methylcellulose. After 24 hours, serial dilutions of MRTX1133, with or without Cetuximab, were added to the tumor spheroid culture medium (1:1 ratio). Cell viability was assessed on day 3 post-treatment by adding 1/10 volume of Alamar Blue reagent (Biosciences, Cat# A016) to the tumor spheroids, followed by incubation for 6–10 hours at 37°C. Fluorescence signals were measured using a fluorescence plate reader (Bio-Tek EPOCH; excitation at 570 nm, emission at 600 nm) to estimate the relative number of viable cells. For organoid models, well-grown organoids were dissociated and seeded into 96-well plates (6 μL organoid suspension with 50% Matrigel per well). After 72 hours of drug treatment, cell viability was assessed using the CellTiter-Glo^®^ 3D Cell Viability Assay (Promega, Cat# G9681) to evaluate the cytotoxic effects of different treatments.

#### Lineage tracing of Paneth-like cells

For lineage tracing using Cre-LoxP recombination system,^67^ LS174T cells were transduced with DEFA5 promoter-driven CreERT2 and CMV promoter-driven LoxP-dsRed-STOP-LoxP-GFP constructs through lentiviral infection, selected by puromycin and blasticidin, respectively. The human DEFA5 promoter region was cloned by PCR and inserted into pCDH-CMV-EF1-puro lentiviral vector to replace the original CMV promoter. The Cre-ERT2 coding sequence (PCR amplified from the pCAG-ERT2CreERT2, Addgene Cat# 13777) was subsequently inserted downstream of the DEFA5 promoter to generate the DEFA5 promoter-driven Cre-ERT2 plasmid. The CMV promoter-driven LoxP-dsRed-STOP-LoxP-eGFP construct was created by inserting the LoxP-dsRed-STOP-LoxP-eGFP DNA fragment (PCR amplified from the pMSCV- LoxP-dsRed-STOP-LoxP-eGFP -Puro-WPRE, Addgene Cat# 32702) into the pCDH-CMV-EF1-blasticidin vector. All plasmids were sequenced to confirm the correct insertion and orientation of the constructs.

For lineage tracing using CRISPR-Cas9 based gene editing strategy, single guide RNA (sgRNA) and homology directed repair (HDR) donor plasmids were designed as previously described.^68^ The sgRNA was designed to induce a double-stranded break (DSB) directly upstream of the DEFA5 stop codon. The HDR knock-in construct was based on the pCDH plasmid backbone with the CMV expression cassette removed. Homology arms ~500bp in length 5’ and 3’ flanking the end of DEFA5 stop codon were amplified from LS174T cells’ genomic DNA. A copGFP fluorescent reporter flanked by 500bp homology arms was cloned into the HDR construct by seamless cloning (Abclonal, Cat# RK21020). The sgRNA-Cas9 plasmid and HDR constructs were co-transfected into LS174T cells. Then puromycin selection was conducted to kill non-transfected cells. Clones derived from single cell expansion were validated by sequencing using primers specifically detecting at the targeted locus. Primers are listed in Table S3.

For specifically ablate Paneth-like cells, LS174T cells were transduced with a lentiviral construct carrying the DEFA5 promoter-driven iCasp9-IRES-eGFP cassette and selected with puromycin. The target ablation plasmid was generated by cloning the iCasp9-IRES-eGFP fragment from the pMSCV-F-del Casp9-IRES-eGFP plasmid into the DEFA5 promoter region of the pCDH-Puro backbone lacking the CMV promoter. The pMSCV-F-del Casp9-IRES-eGFP plasmid was a gift from Yan Yan. To evaluate the efficacy of AP1903-mediated targeted ablation of Paneth-like cells in 3D spheroids, collected spheroids were stained with Hoechst 33342 (1 μg/mL, Invitrogen, Cat# H3570) and propidium iodide (PI, 1 μg/mL) for 15 minutes, followed by confocal microscopy imaging.

#### Construction of human TFome CRISPR sgRNA library

A total of 1,739 transcription factors, known to directly or indirectly interact with DNA, were sourced from the AnimalTFDB (Nucleic Acids Res. 2015 Jan;43(Database issue): D76–81) and the JASPAR database (https://doi.org/10.1093/nar/gkz1001). For each gene, 10 single guide RNAs (sgRNAs) were designed. The target sequences for sgRNA were retrieved from three established CRISPR libraries: Human Activity-Optimized CRISPR Knockout Library (from David Sabatini, Eric Lander’s labs), Human CRISPR Knockout Pooled Library (GeCKO v2, from Feng Zhang’s lab), and Toronto KnockOut (TKO) CRISPR Library (from Jason Moffat’s lab). For genes with fewer than 10 sgRNAs available from reference libraries, additional sgRNAs were designed using the Graphical User Interface for DNA Editing Screens (GUIDES) software. The sgRNA oligonucleotides, including the flanking adapters, were synthesized through the Syno^®^ 3.0-oligo pool synthesis platform (Synbio Technologies). These oligo pools were then amplified and inserted into the lenti-CRISPR v2 vector via Gibson assembly, according to the manufacturer’s protocol.

#### *In vitro* CRISPR screen in 3D cancer spheroids

The Human TFome CRISPR sgRNA library plasmids were transfected into HEK-293T cells to generate lentiviral pools, which were subsequently used to infect LS174T cells at a multiplicity of infection (MOI) of 0.2–0.3. After 48 hours, cells were selected with puromycin for 7 days. Cell aliquots were collected as T0 samples and frozen at −80°C. The remaining cells were cultured as 3D spheroids and treated with either DMSO or MRTX1133 at a concentration of 2nM (IC20) and Cetuximab (25nM) for 14 days. To ensure the complexity of the library, the screen was performed with approximately 1,000× cell number coverage per sgRNA in each biological replicate. After 14 days, genomic DNA was extracted and sgRNA cassettes were amplified by PCR. The abundance of sgRNA was quantified through high throughput sequencing and analyzed with MAGeCKFlute.

#### Plasmid construction and lentivirus infection

The sgRNAs targeting human SMAD1 and FGFR3 were cloned into the pLentiCRISPR v2 vector using BsmbI restriction sites. The shRNAs targeting human SMAD4, SMAD5 and SMAD9 were cloned into pGreenPuro-shRNA vector using BamHI and EcoRI restriction sites. The sequences of all sgRNAs and shRNAs primer pairs are shown in Table S4. The SMAD1 overexpression construct was generated by amplifying the full-length SMAD1 cDNA via PCR and cloning it into the pCDH-CMV-MCS-EF1-Puro vector.

To generate lentivirus, HEK-293T cells were co-transfected with either pLentiCRISPR v2, pGreenPuro-shRNA or pCDH plasmid, along with packaging plasmids PSPAX2 and PMD2.G, using polyethyleneimine (PEI) as the transfection reagent. The lentiviral supernatants were collected and filtered through 0.45-μm filters to remove cellular debris. To transduce LS174T and LS180 cells, the filtered lentiviral supernatants were added to the cells in the presence of Polybrene (Sigma). Stable cell lines were selected using 2 μg/ml puromycin or 5 μg/ml blasticidin.

#### Immunohistochemistry and immunofluorescence of tissue samples

Tissue samples from iKAP orthotopic tumors, CDX, and PDX models were harvested, fixed in 4% paraformaldehyde for 24 hours, and subsequently embedded in paraffin. The tissues were sectioned into 3-μm thick slices for further analysis. The slides were baked overnight at 63°C, then deparaffinized three times with xylene, each for 10 minutes. Gradient hydration was performed with ethanol solutions (100%, 95%, 85%, and 75%) and distilled water, with each step lasting 5 minutes. After hydration, endogenous peroxidase activity was quenched by incubating the slides in 3% hydrogen peroxide for 30 minutes. Antigen retrieval was performed using a steamer, and the sections were permeabilized with 0.5% Triton X-100 for 20 minutes. Blocking was carried out with 10% goat serum for 30 minutes. Sections were then incubated with primary antibodies overnight at 4°C. The primary antibodies included: anti-DEFA5 (Novus Biologicals, Cat# NB110–60002, 1:2000) for human samples, anti-DEFA5 (Abbexa, Cat# abx176113, 1:2000) for mouse samples, anti-panCK (CST, Cat# 4523, 1:200) for human samples, anti-panCK (Abcam, Cat# ab7753, 1:1000) for mouse samples, anti-CDX2 (CST, Cat# D11D10, 1:1000), anti-CK20 (CST, Cat# D9Z1Z, 1:1000), anti-Ki-67 (Abcam, Cat# ab16667, 1:200), cleaved caspase3 (CST, Cat# 9661, 1:200), FGFR3 (phospho Y724) (Abcam, Cat# ab155960, 1:400) and LYZ (Abcam, Cat# ab108508, 1:1000).

For immunohistochemistry, following primary antibody incubation, sections were incubated with the appropriate secondary antibodies for 30 minutes, followed by color development using the DAB substrate kit (ZSGB-bio, Cat# PV-6000D). Sections were counterstained with hematoxylin and mounted.

For multiplex immunofluorescence, the Goat Anti-Mouse/Rabbit Multiplex IHC Detection Kit (Zenbio, Cat# 18003) was used following the manufacturer’s protocol. After primary antibody incubation, slides were incubated with HRP-conjugated secondary antibodies at room temperature in the dark for 1 hour. Tyramide signal amplification (TSA) was performed with TSA-520, TSA-570, or TSA-690 dyes for 10 minutes at room temperature. Heat-induced epitope retrieval (HIER) was applied after each TSA cycle to enable successive rounds of multiplexed staining with different primary antibodies. Slides were then mounted with Anti-fade Mounting Medium containing DAPI (Beyotime, Cat# P0131), and images were acquired using a fluorescent scanner for analysis.

#### Immunofluorescence of CRC organoids and 3D spheroids

Organoids were retrieved from Matrigel using Cell Recovery Solution (Corning, Cat# 354253) for 30 minutes on ice. Organoids and 3D tumor spheroids derived from colorectal cancer (CRC) cell lines were collected in centrifuge tubes and fixed in 4% paraformaldehyde for 30 minutes. Following fixation, the samples were permeabilized with 1 mL of 0.3% Triton X-100 in PBS at room temperature for 15 minutes. Blocking was performed with 5% bovine serum albumin (BSA) in PBS for 30 minutes at room temperature. The spheroids were then incubated with primary antibodies overnight at 4°C. The primary antibodies used were as follows: anti-EPCAM (ABclonal, Cat# A19301, 1:200), anti-SMAD1 (Proteintech, Cat# 10429–1-A, 1:1000) and anti-DEFA5 (Novus Biologicals, Cat# NB110–60002, 1:1000). After primary antibody incubation, spheroids were incubated with the secondary fluorescent antibody for 1 hour at room temperature. The spheroids were collected and spread onto slides, and the nuclei were stained with DAPI. Finally, the slides were mounted for observation with laser-scanning confocal microscopy (Zeiss LSM 980).

#### Western blots and antibodies

For Western blot analysis, cells were lysed on ice using RIPA buffer (Beyotime, Cat# P0013) supplemented with protease inhibitor cocktail (MCE, Cat# HY-K0010). Protein concentrations were quantified using the BCA Protein Assay Kit (Beyotime, Cat# P0009). The primary antibodies used for protein detection included anti-SMAD1 (Proteintech, Cat# 10429–1-A), anti-FGFR3 (santa cruz, Cat# sc390423), anti-FGFR3 (phospho Y724) (Abcam, Cat# ab155960), anti-p217/p221-MEK1/2 (p-MEK1/2) (CST, Cat# 9154), anti-p202/p204-ERK1/2 (p-ERK1/2) (CST, Cat# 4370), anti-ERK1/2 (CST, Cat# 4696), anti-SMAD4 (CST, Cat# 46535), anti-SMAD5 (Proteintech, Cat# 12167–1-AP), anti-SMAD9 (Proteintech, Cat# 16397–1-AP), NRAS (santa cruz, Cat# sc-31), HRAS (santa cruz, Cat# sc-53959) and anti-GAPDH (santa cruz, Cat# sc32233).

#### Quantitative Real Time PCR (RT-qPCR)

Total RNA was isolated from the cells using the EZ-press RNA Purification Kit (EZBioscience, Cat# B0004D), and complementary DNA (cDNA) was synthesized with PrimeScriptTM RT Master Mix (Takara, Cat# RR047A). Quantitative PCR was carried out with SYBR Green PCR Master Mix (Vazyme, Cat# Q711–02). The relative expression levels of target genes were calculated using the comparative 2^−ΔΔCt^ method, with normalization first to the endogenous control GAPDH and subsequently to the designated control group. Each sample was analyzed in triplicate. The primer sequences used in the PCR are listed in Table S5.

#### ChIP-PCR

ChIP was carried out following a previously established protocol with slight modifications.^69^ In brief, cells were cross-linked with 1.42% formaldehyde for 15 minutes at room temperature. Cross-linking was quenched by adding 125 mM glycine for 5 minutes. The cells were then washed twice with cold PBS and collected by scraping. The cell pellets were resuspended in 500 μL of lysis buffer containing 50 mM Hepes/KOH (pH 7.5), 140 mM NaCl, 1 mM EDTA, 1% Triton X-100, 0.1% Na-deoxycholate, and protease inhibitors. The cell lysates were sonicated using a Covaris E220 (Peak incident power 105w, Duty factor 10%, Cycles per burst 200, Treatment time 110s). Following sonication, clarified samples were collected by centrifugation. 50μL of the supernatants were reserved as input. The remaining supernatants were incubated with the anti-V5 agarose affinity gel beads overnight at 4°C (sigma, Cat# A7345). After incubation, the beads were washed twice with Low Salt Wash buffer, High Salt Wash buffer, Tris/LiCl Wash buffer and TE buffer, then resuspended in 50μL elution buffer (10mM Tris HCl, pH 8.0; 10mM EDTA; 150mM NaCl; 5mM DTT and 1% SDS). The samples were boiled for 10 minutes and then centrifuged at 4°C for 1 minute. The supernatants were transferred to new tubes. An additional 50μL of elution buffer was added to the beads, followed by vortexing for 10 seconds and another centrifugation step to pellet the beads. The supernatants from both steps were pooled and used as templates for subsequent qPCR analysis, primers are listed in Table S5.

#### RNA-seq and data analysis

Colorectal tumors from the vehicle and treatment groups of the iKAP model were collected for bulk RNA sequencing (RNA-seq). Similarly, RNA-seq was performed on LS174T cells with or without SMAD1 knockout treated with either DMSO or MRTX1133 and Cetuximab for 72h. RNA-seq data were aligned to the mouse and human reference genomes (mm9 and hg38, respectively) using STAR software (version 2.4.2a). Read counts for each gene were quantified based on the Ensembl GRCm38.80 and GRCh38 annotations. Differential expression analysis was performed using DESeq2 (version 1.10.0) in R (version 4.2.1) with a threshold of log2 fold-change (LFC) >1 and false discovery rate (FDR) < 0.05. Gene set enrichment analysis (GSEA) was conducted to identify enriched pathways and biological processes. P value <0.05 was set as statistically significant based on LFC rank from DESeq2 analysis.

#### Single cell RNA-seq data analysis

*KRAS-G12D* mutant PDO single-cell sequencing data were generated with 10x Genomics for sequential samples (0d, 2d, 3d, 7d, and 14d) and the Cell Ranger raw counts were imported into Seurat (version 5.3.0) for sample merging and quality assessment. Only cells with RNA features between 250 and 7000 and mitochondrial RNA < 15% were retained for further analysis. For dimension reduction, raw counts were transformed to pairwise ratios over each of 50 control gene modules with FeatPairs (version 1.0.0) (https://github.com/FangZY-Lab/FeatPairs), filtered to 2000 highly variable features, reduced to first 25 principal components with the implicitly restarted Lanczos method on scaled data in irlba (version 2.3.5.1), and combined for Euclidean distance-based UMAP learning with uwot (version 0.2.3), k-nearest neighbor graph (k = 20) construction and Leiden algorithm-based clustering (resolution = 1.2e-4) with monocle3 (version 1.4.26). Small isolated spurious clusters (size < 250) were considered as technical noises and only high-quality communities were kept for subsequent trajectory learning (minimal_branch_len = 40, ncenter = 1000, nn.k = 45). Cells were ordered by setting two principal nodes at 0d as roots. Cell cycle phasing was performed with Seurat and the internal cell cycle signature genes. Gene signature scoring for each cell was performed with UCell (version 2.12.0).

#### Whole exome sequencing data processing

We performed whole-exome sequencing (WES) on post-treatment samples from Patient #1 (SYSUCC) and on tumors from the KRAS G12D PDX #1 model treated with either vehicle or MRTX1133 plus Cetuximab. Raw sequencing reads were processed with TrimGalore (version 0.6.7). Sequenced reads were then aligned to the reference genome assemble hg38 using bwa-mem (version 0.7.17-r1188), and subsequent processing including sorting reads, removing duplicates and base quality score recalibration were performed using samtools (version 1.9)^70^ and GATK Toolkit (version 4.2.6.1).^71^ Then GATK somatic short variants discovery workflow was applied to call somatic mutations (single nucleotide variants (SNVs) and indels). The exon regions of target genes were extracted and merged from gtf file in gencode (version 45). Vcf2maf (version 1.6.19) was used to annotate somatic mutations with emsembl-vep (version 105) and convert annotated vcf to maf file.

### QUANTIFICATION AND STATISTICAL ANALYSIS

Data are presented as mean ± SD or mean ± SEM, as indicated. For comparisons between two groups, statistical significance was determined using a two-tailed Student’s t-test. For comparisons among more than two groups, one-way ANOVA with Dunnett’s or Bonferroni’s multiple comparisons test was applied, as appropriate. For repeated measures, such as tumor growth curves, two-way ANOVA was used. Statistical analyses were performed using Prism (GraphPad Software). For *in vitro* experiments, samples were randomly assigned to vehicle or treatment groups, and each experiment was independently repeated at least three times with consistent results. For *in vivo* studies, mice were randomized into treatment or control groups, and sample sizes are indicated in figure legends.

## Supplementary Material

1

Supplemental information can be found online at https://doi.org/10.1016/j.ccell.2025.10.010.

## Figures and Tables

**Table:** KEY RESOURCES TABLE

REAGENT or RESOURCE	SOURCE	IDENTIFIER
Antibodies		
DEFA5 (human)	Novus Biologicals	Cat# NB110-60002; RRID: AB_905358
DEFA5 (mouse)	Abbexa	Cat# abx176113; RRID: AB_3096419
SMAD1	Proteintech	Cat# 10429-1-AP; RRID: AB_2247334
EpCAM	ABclonal	Cat# A19301; RRID: AB_2862637
panCK (human)	Cell Signaling Technology	Cat# 4523; RRID: AB_836889
panCK (mouse)	Abcam	Cat# ab7753; RRID: AB_306047
CDX2	Cell Signaling Technology	Cat# 84638; RRID: AB_2943240
CK20	Cell Signaling Technology	Cat# 13063; RRID: AB_3101888
Ki-67	Abcam	Cat# ab16667; RRID: AB_302459
cleaved caspase3	Cell Signaling Technology	Cat# 9661; RRID: AB_2341188
LYZ	Abcam	Cat# ab108508; RRID: AB_10861277
FGFR3	santa cruz	Cat# sc390423; RRID: AB_631511
FGFR3 (phospho Y724)	Abcam	Cat# ab155960; RRID: AB_3095625
p217/p221-MEK1/2	Cell Signaling Technology	Cat# 9154; RRID: AB_2138017
p202/p204-ERK1/2	Cell Signaling Technology	Cat# 4370; RRID: AB_2315112
ERK1/2	Cell Signaling Technology	Cat# 4696; RRID: AB_390780
SMAD5	Proteintech	Cat# 12167-1-AP; RRID: AB_2286502
SMAD9	Proteintech	Cat# 16397-1-AP; RRID: AB_2270847
SMAD4	Cell Signaling Technology	Cat# 46535; RRID: AB_2736998
NRAS	santa cruz	Cat# sc-31; RRID: AB_628041
HRAS	santa cruz	Cat# sc-53959; RRID: AB_1124799
GAPDH	santa cruz	Cat# sc32233; RRID: AB_627679
Biological samples		
Human CRC patient #1 paraffin sections	Sun Yat-sen University Cancer Center	Table S2
Human CRC patient #2 paraffin sections	Memorial Sloan Kettering Cancer Center	Table S2
Chemicals, peptides, and recombinant proteins		
MRTX1133	TargetMol	Cat# T9303
Cetuximab	Merck	Cat# 10736
Sotorasib	Bidepharm	Cat# BD01124336
Infigratinib	TargetMol	Cat# T1975
Futibatinib	TargetMol	Cat# T5044
4-Hydroxytamoxifen	Sigma Aldrich	Cat# H7904
Doxycycline	Macklin	Cat# 10592-13-9
Poly-HEMA	Sigma Aldrich	Cat# P3932
methycellulose	Sigma Aldrich	Cat# M0512
type II collagenase	GIBCO	Cat# 17101015
type II dispase	Sigma Aldrich	Cat# D4693
Advanced DMEM/F12	Invitrogen	Cat# 12634-028
Glutamax	Invitrogen	Cat# 35050-079
HEPES	Invitrogen	Cat# 15630-056
Nicotinamide	Sigma Aldrich	Cat# N0636-500G
N-acetyl-cysteine	Sigma Aldrich	Cat# A9165-5G
N2 supplement	Fisher Scientific	Cat# 17502048
Gastrin	Sigma Aldrich	Cat# G9145
A83-01	Tocris	Cat# 2939
B27	Fisher Scientific	Cat# 17504-044
FGF2	R & D Systems	Cat# 233-FB-025
Noggin	Peprotech Inc.	Cat# 120-10C
Y-27632	Abmole	Cat# 12-541-0
SB202190	Sigma Aldrich	Cat# S7067-25MG
primocin	invivo gene	Cat# ant-pm-1
TrypLE	GIBCO	Cat# 12605010
Cell Recovery Solution	Corning	Cat# 354253
Matrigel	Corning	Cat# 354234
FGF20	MCE	Cat# HY-P700062AF
FGF23	MCE	Cat# HY-P7013
BMP2	TargetMol	Cat# TMPH-01014
BMP4	TargetMol	Cat# TMPY-06842
TGF-β	MCE	Cat# HY-P7118
Alamar Blue	Biosciences	Cat# A016
Hoechst 33342	Invitrogen	Cat# H3570
Antifade Mounting Medium with DAPI	Beyotime	Cat# P0131
RIPA buffer	Beyotime	Cat# P0013
protease inhibitor cocktail	MCE	Cat# HY-K0010
anti-V5 agarose affinity gel beads	sigma	Cat# A7345
Critical commercial assays		
EZ-press RNA Purification Kit	EZBioscience	Cat# B0004D
PrimeScriptTM RT Master Mix	Takara	Cat# RR047A
SYBR Green PCR Master Mix	Vazyme	Cat# Q711-02
2X MultiF Seamless Assembly Mix	Abclonal	Cat# RK21020
CellTiter-Glo^®^ 3D Cell Viability Assay	Promega	Cat# G9681
BCA Protein Assay Kit	Beyotime	Cat# P0009
DAB substrate kit	ZSGB-bio	Cat# PV-6000D
Goat Anti-Mouse/Rabbit Multiplex IHC Detection Kit	Zenbio	Cat# 18003
Deposited data		
Public bulk RNA-seq data from CRC cohort 1	Freeman et al.^47^	GEO: GSE17536
Public bulk RNA-seq data from CRC cohort 2	Li et al.^48^	OMIX: OMIX004653, OMIX004655
TCGA COAD cohort	XENA data portal^54^	http://xena.ucsc.edu/
bulk RNA-seq for iKAP mice	This study	GSA: CRA031224
bulk RNA-seq for LS174T cell line	This study	GSA: HRA010461
WES data	This study	GSA: HRA012755, HRA012707, HRA013736
scRNA-seq data	This study	OMIX: OMIX011372
CRISPR-Cas9 screen data	This study	GSA: HRA013731
Experimental models: Cell lines		
Human: LS174T	ATCC	Cat# CL-188
Human: LS180	ATCC	Cat# CL-187
Human: RW7213	Sandra Misale laboratory	N/A
Human: SW1463	Sandra Misale laboratory	N/A
Human organoid: KRAS G12C PDO	This study	N/A
Human organoid: KRAS G12D PDO#1	This study	N/A
Human organoid: KRAS G12D PDO#2	This study	N/A
Experimental models: Organisms/strains		
Mouse: iKAP GEMM *(tet-O-LSL-Kras*^*G12D*^*; Apc*^*lox/lox*^; *Trp53*^*lox/lox*^*; Villin-cre-ERT)*	Wenting Liao laboratory	N/A
Mouse: NSG	Guangdong Medical Laboratory Animal Center	N/A
Mouse: BALB/c nude	Gempharmatech-GD	Cat# D000521
Oligonucleotides		
Primers for lineage tracing	This study	Table S3
Primers for shRNA/sgRNA	This study	Table S4
Primers for qRT-PCR	This study	Table S5
Recombinant DNA		
DEFA5-pro-Cre-ERT2	This study	N/A
CMV-Loxp-dsRed-STOP-Loxp-eGFP	This study	N/A
sgRNA-hDEFA5-knockin	This study	N/A
hDEFA5-knockin-CopGFP-HDR-donor	This study	N/A
DEFA5-pro-iCasp9-IRES-eGFP	This study	N/A
lentiCRISPR v2 sg-CTRL-mcherry	This study	N/A
lentiCRISPR v2 sg-SMAD1-EGFP-1# and 2#	This study	N/A
lentiCRISPR v2 sg-FGFR3-EGFP-1# and 2#	This study	N/A
pCDH-SMAD1-puro	This study	N/A
pGreenPuro-shSMAD4-1# and 2#	This study	N/A
pGreenPuro-shSMAD5-1# and 2#	This study	N/A
pGreenPuro-shSMAD9-1# and 2#	This study	N/A
Software and algorithms		
Graphpadprism9	GraphpadSoftware	https://www.graphpad.com
Rstudio	Rstudio	https://rstudio.com/
R (version 4.2.1)	CRAN	https://www.r-project.org/
STAR (version 2.5.1b)	Github	https://github.com/alexdobin/STAR
RSEM (version 1.3.3)	Github	https://github.com/deweylab/RSEM
DESeq2 (version 1.10.0)	Bioconductor	https://bioconductor.org/packages/release/bioc/html/DESeq2.html
GSVA (version 1.49.1)	Bioconductor	https://bioconductor.org/packages/release/bioc/html/GSVA.html
ggplot2 (version 3.3.5)	CRAN	https://cran.r-project.org/web/packages/%20ggplot2/index.html
ggpubr (version 0.6.0)	CRAN	https://cran.r-project.org/package=ggpubr
ggsignif (version 0.6.4)	CRAN	https://cran.r-project.org/web/packages/ggsignif/index.html
monocle3 (version 1.4.26)	Github	https://github.com/cole-trapnell-lab/monocle3
CellRanger (version 7)	10x Genomics	https://10xgenomics.com
Seurat (version 5.3.0)	CRAN	https://cran.r-project.org/web/packages/Seurat/index.html
FeatPairs (version 1.0.0)	Github	https://github.com/FangZY-Lab/FeatPairs
Uwot (version 0.2.3)	CRAN	https://cran.r-project.org/web/packages/uwot/index.html
UCell (version 2.12.0)	GitHub	GitHub - carmonalab/UCell: Gene set scoring for single-cell data
GATK (version 4.2.6.1)	broadinstitute	https://software.broadinstitute.org/gatk/
BWA-mem (version 0.7.17-r1188)	Github	https://github.com/bwa-mem2/bwa-mem2
Samtools (version 1.9)	sourceforge	http://samtools.sourceforge.net/
Vcf2maf (version 1.6.19)	Github	https://github.com/mskcc/vcf2maf

## Data Availability

Further information and requests for resources and reagents should be directed to and will be fulfilled by the lead contact, Yijun Gao (gaoyj@sysucc.org.cn). Plasmids and PDOs generated during this study are available upon request from the lead contact with a completed materials transfer agreement. All sequencing data generated in this study have been deposited in the National Genomics Data Center and are publicly accessible. The accession numbers are as follows: RNA-seq data from iKAP mice and LS174T cell lines have been deposited in the Genome Sequence Archive (GSA, https://ngdc.cncb.ac.cn/gsa/) with accession codes GSA: CRA031224, HRA010461. scRNA-seq preprocessed data have been deposited in Open Archive for Miscellaneous Data (OMIX, https://ngdc.cncb.ac.cn/omix/) with accession code OMIX: OMIX011372. CRISPR-Cas9 screen data have been deposited in the GSA under accession number GSA: HRA013731. Whole-exome sequencing data have been deposited in the GSA under accession numbers GSA: HRA012755, HRA012707, and HRA013736. This study also reanalyzed publicly available bulk RNA-seq data from human CRC cohorts (GEO: GSE17536, OMIX: OMIX004653, OMIX004655) published in prior studies^47,48^ and TCGA colon adenocarcinoma cohort from XENA data portal (http://xena.ucsc.edu/).^54^ This paper does not report original code. Any additional information required to reanalyze the data reported in this paper is available from the lead contact upon request.

## Highlights

- CRC cells undergo a Paneth-like transition to survive dual KRAS and EGFR inhibition
- Targeted ablation of Paneth-like cells markedly enhances dual therapy response
- SMAD1-FGFR3 signaling drives Paneth-like transition and MAPK rebound
- FGFR3 co-targeting overcomes plasticity-driven resistance in *KRAS*-mutant CRC
