# Supplementary material for: Paneth-like transition drives resistance to dual targeting of KRAS and EGFR in colorectal cancer
Source: Cancer Cell. Author manuscript; Available in PMC 2026 Jul 20. (PMC13384395; doi:10.1016/j.ccell.2025.10.010)
Supplement: 1 [file NIHMS2194839-supplement-1.pdf]

Cancer Cell, Volume *44*

Supplemental information

Paneth-like transition drives resistance to dual targeting of KRAS and EGFR in colorectal cancer

Yuetong Zhang, Jiaying Chen, Yong She, Zhaoyuan Fang, Yaxin Zhang, Danyun Ruan, Wenjun Guo, Jianping Liao, Weiping Zhou, Jianpei Lao, Weicheng Fang, Xingyan Pan, Wenfei Kang, Zifeng Wang, Yuanzhong Wu, Rong Deng, Lin Tian, Liqin Wang, Huilin Huang, Jian Zheng, Yan Yan, Hezhe Lu, Ruiping Wang, Rona Yaeger, Qi Zhao, Wenting Liao, Feng Wang, and Yijun Gao

Figure S1

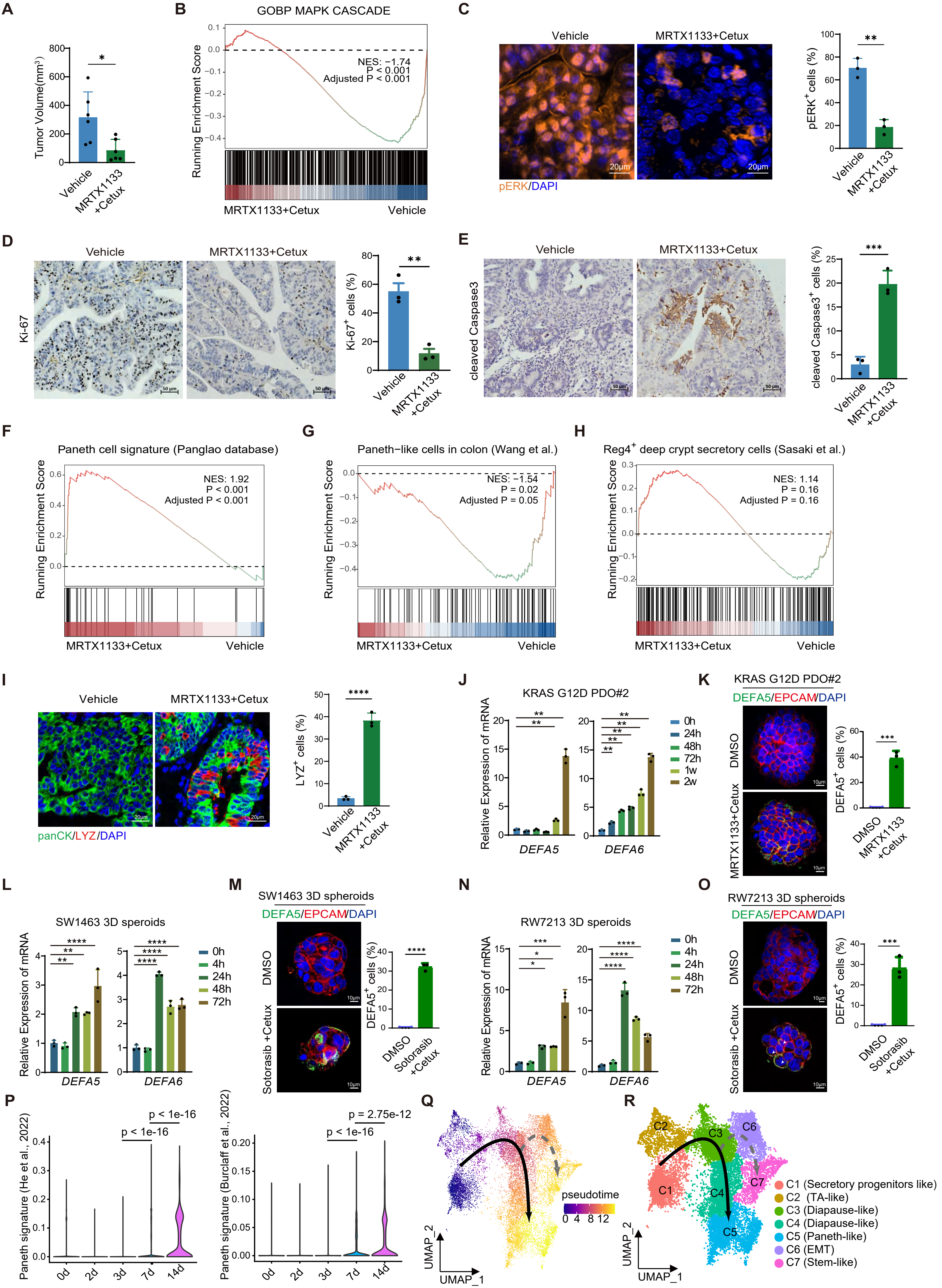
A
Tumor Volume(mm³)
800
600
400
200
0
*
Vehicle
MRTX1133 +Cetux
B
GOBP MAPK CASCADE
Running Enrichment Score
NES: −1.74
P < 0.001
Adjusted P < 0.001
MRTX1133+Cetux
Vehicle
C
Vehicle
MRTX1133+Cetux
20μm
pERK/DAPI
pERK+ cells (%)
**
D
Ki-67
50 μm
Ki-67+ cells (%)
E
cleaved Caspase3
cleaved Caspase3+ cells (%)
***
F
Paneth cell signature (Panglao database)
NES: 1.92
P < 0.001
Adjusted P < 0.001
G
Paneth−like cells in colon (Wang et al.)
NES: −1.54
P = 0.02
Adjusted P = 0.05
H
Reg4+ deep crypt secretory cells (Sasaki et al.)
NES: 1.14
P = 0.16
Adjusted P = 0.16
I
panCK/LYZ/DAPI
LYZ+ cells (%)
****
J
KRAS G12D PDO#2
Relative Expression of mRNA
DEFA5
DEFA6
0h
24h
48h
72h
1w
2w
K
KRAS G12D PDO#2
DEFA5/EPCAM/DAPI
DMSO
MRTX1133+Cetux
10μm
DEFA5+ cells (%)
L
SW1463 3D speroids
0h
4h
24h
48h
72h
M
SW1463 3D spheroids
DEFA5/EPCAM/DAPI
Sotorasib +Cetux
N
RW7213 3D speroids
O
RW7213 3D spheroids
P
Paneth signature (He et al., 2022)
Paneth signature (Burclaff et al., 2022)
p < 1e-16
p = 2.75e-12
0d
2d
3d
7d
14d
Q
pseudotime
UMAP_1
UMAP_2
R
C1 (Secretory progenitors like)
C2 (TA-like)
C3 (Diapause-like)
C4 (Diapause-like)
C5 (Paneth-like)
C6 (EMT)
C7 (Stem-like)

Supplementary Figure 1. Enrichment of Paneth-like cell state in residual CRC lesions following KRAS-EGFR inhibition. Related to Figure 1. (A) Quantification of tumor volume of iKAP mice treated with vehicle, MRTX1133 + Cetuximab. Data are presented as mean ± SD (n = 6), analyzed by two-tailed Student's t-test, $^{*}p < 0.05$. **(B)** GSEA plot showing negative modulation of the GO term "GOBP_MAPK_CASCADE" in MRTX1133+Cetuximab-treated iKAP tumors compared with vehicle-treated tumors. Statistical significance was determined by permutation testing with multiple hypothesis correction, using the normalized enrichment score (NES). **(C)** Immunofluorescence staining of pERK in iKAP tumors treated with vehicle or MRTX1133+ Cetuximab (left), with corresponding statistical analysis (right). Scale bar = 20μm. Data are presented as mean ± SD (n = 3 mice) and analyzed by a two-tailed t-test, $^{**}p < 0.01$. **(D-E)** Immunohistochemical staining of Ki-67 (D) and cleaved caspase3 (E) in iKAP tumors treated with vehicle or MRTX1133+ Cetuximab (left), with corresponding statistical analysis (right). Scale bar= 50μm. Data are presented as mean ± SD (n = 3 mice) and analyzed by a two-tailed t-test, $^{**}p < 0.01$, $^{***}p < 0.001$. **(F)** GSEA plot denoting that MRTX1133 + Cetuximab treatment upregulated the expression of Paneth cell signature genes derived from the Panglao database in iKAP tumors. Statistical significance was determined by permutation testing with multiple hypothesis correction, using the normalized enrichment score (NES). **(G)** GSEA plot showing a downward trend for Paneth-like cells of colon signature in iKAP tumors treated with MRTX1133 + Cetuximab. Statistical significance was determined by permutation testing with multiple hypothesis correction, using the normalized enrichment score (NES). **(H)** GSEA plot showing no enrichment for Reg4+ deep crypt secretory cell signature in iKAP tumors treated with MRTX1133 + Cetuximab. Statistical significance was determined by permutation testing with multiple hypothesis correction, using the normalized enrichment score (NES). **(I)** Immunofluorescence staining of LYZ and panCK in iKAP tumors treated with vehicle or MRTX1133+ Cetuximab (left), with corresponding statistical analysis (right). Scale bar = 20μm. Data are presented as mean ± SD (n = 3 mice) and analyzed by a two-tailed t-test, $^{****}p < 0.0001$. **(J, L, N)** RT-qPCR analysis of Paneth cell marker genes *DEFA5* and *DEFA6* in KRAS G12D PDO#2 (J), SW1463 3D spheroids (L) and RW7213 3D spheroids (N) over a time course following treatment with MRTX1133(100nM) or Sotorasib (100nM) combined with Cetuximab (25nM), normalized to the reference gene *GAPDH*. Data are presented as mean ± SD (n = 3) and analyzed by one-way ANOVA test, $^{*}p < 0.05$, $^{**}p < 0.01$, $^{***}p < 0.001$, $^{****}p < 0.0001$. **(K, M, O)** Representative images and quantification of immunofluorescence staining of DEFA5, EPCAM and DAPI in KRAS G12D PDO#2 (K), SW1463 3D spheroids (M) and RW7213 3D spheroids (O) treated with DMSO or MRTX1133 (100nM) or Sotorasib (100nM) combined with Cetuximab (25nM) for 2 wks or 72h. Scale bar = 10μm. Data are presented as mean ± SD (n = 3) and analyzed by two-tailed t-test, $^{***}p < 0.001$, $^{****}p < 0.0001$. **(P)** Temporal distribution of Paneth cell signatures along the experimental sampling time points. **(Q)** UMAP embedding of cells overlaying with the trajectory of cell state transitions inferred by Monocle. **(R)** UMAP embedding of cells colored according to their pseudotime inferred by Monocle.

Figure S2

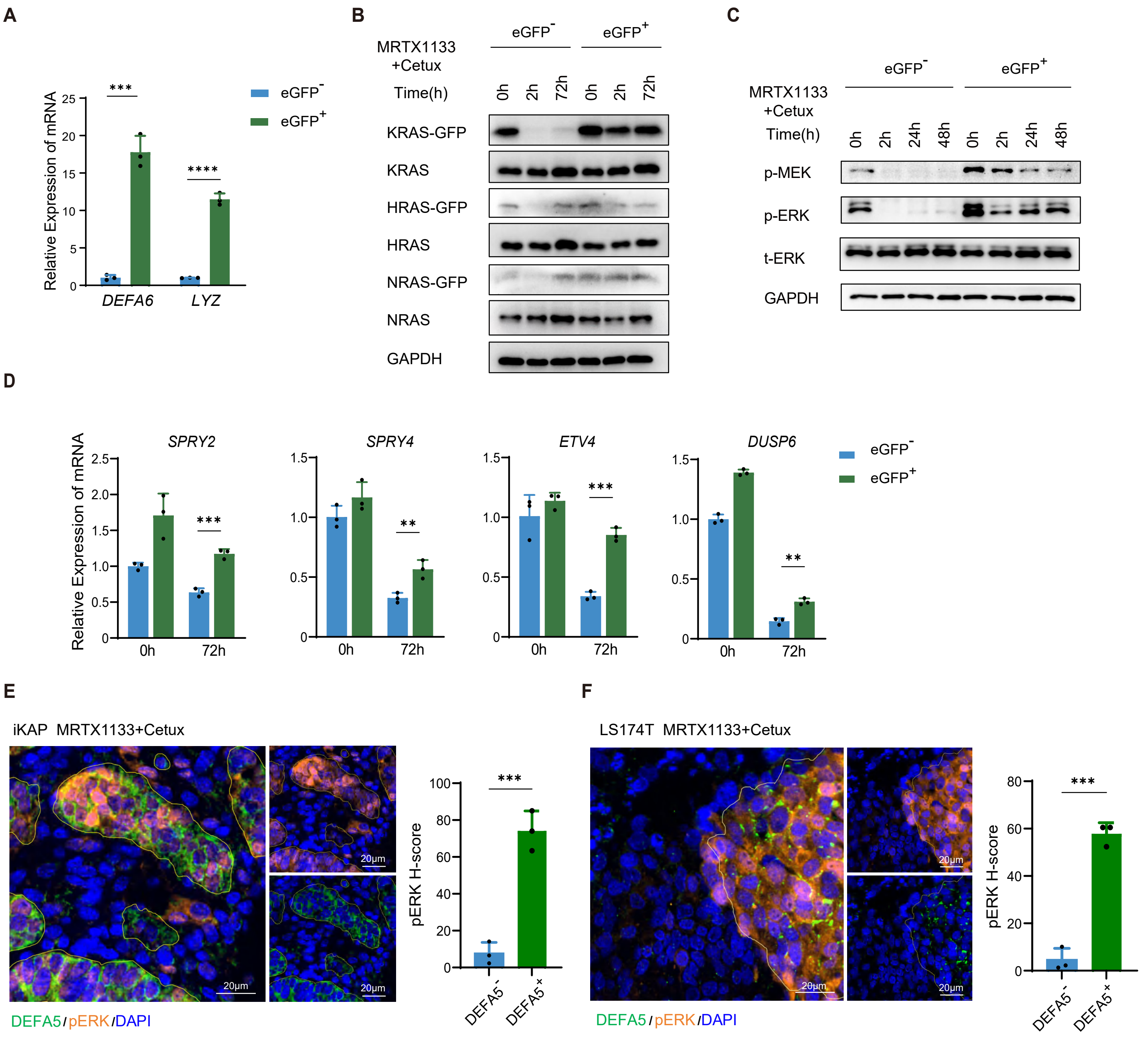

Supplementary Figure 2. Paneth-like cells emerge via trans-differentiation in response to combined KRAS and EGFR inhibition. Related to Figure 2. (A) RT-qPCR analysis of Paneth cell marker genes, *DEFA6* and *LYZ*, in sorted eGFP$^-$ and eGFP$^+$ CRC cells, normalized to GAPDH and presented relative to eGFP$^-$ cells . Data are presented as mean ± SD (n = 3) and analyzed by two-tailed t-test, ***p < 0.001, ****p < 0.0001. **(B)** Western blot analysis of KRAS-, HRAS-, and NRAS-GTP and their respective RAS inputs from eGFP$^+$ Paneth-like cells and eGFP$^-$ cells treated with MRTX1133 (100nM) + Cetuximab (25nM) over time. **(C)** Western blot analysis of MAPK signaling response in MRTX1133 (100nM) + Cetuximab (25nM) treated eGFP$^-$ and eGFP$^+$ CRC cells. GAPDH is used as loading control. **(D)** RT-qPCR analysis of MAPK signaling output genes, including *SPRY2*, *SPRY4*, *ETV4* and *DUSP6*, in sorted eGFP$^-$ and eGFP$^+$ CRC cells treated with MRTX-1133(100nM)+ Cetuximab (25nM) for 72h, normalized to the reference gene *GAPDH*. Data are presented as mean ± SD (n = 3) and analyzed by two-tailed t-test, **p < 0.01, ***p < 0.001. **(E-F)** Representative images showing pERK (orange) and DEFA5 (green) immunofluorescence in iKAP tumors (E) and LS174T xenografts (F) treated with MRTX1133+Cetux for 2weeks (left), along with corresponding quantification of pERK staining intensity in pERK$^+$ and DEFA5$^+$ vs. pERK$^+$ and DEFA5$^-$ cells (right). Scale bar = 20μm. Data are presented as mean ± SD (n = 3), analyzed by two-tailed t-test, ***p < 0.001.

Figure S3

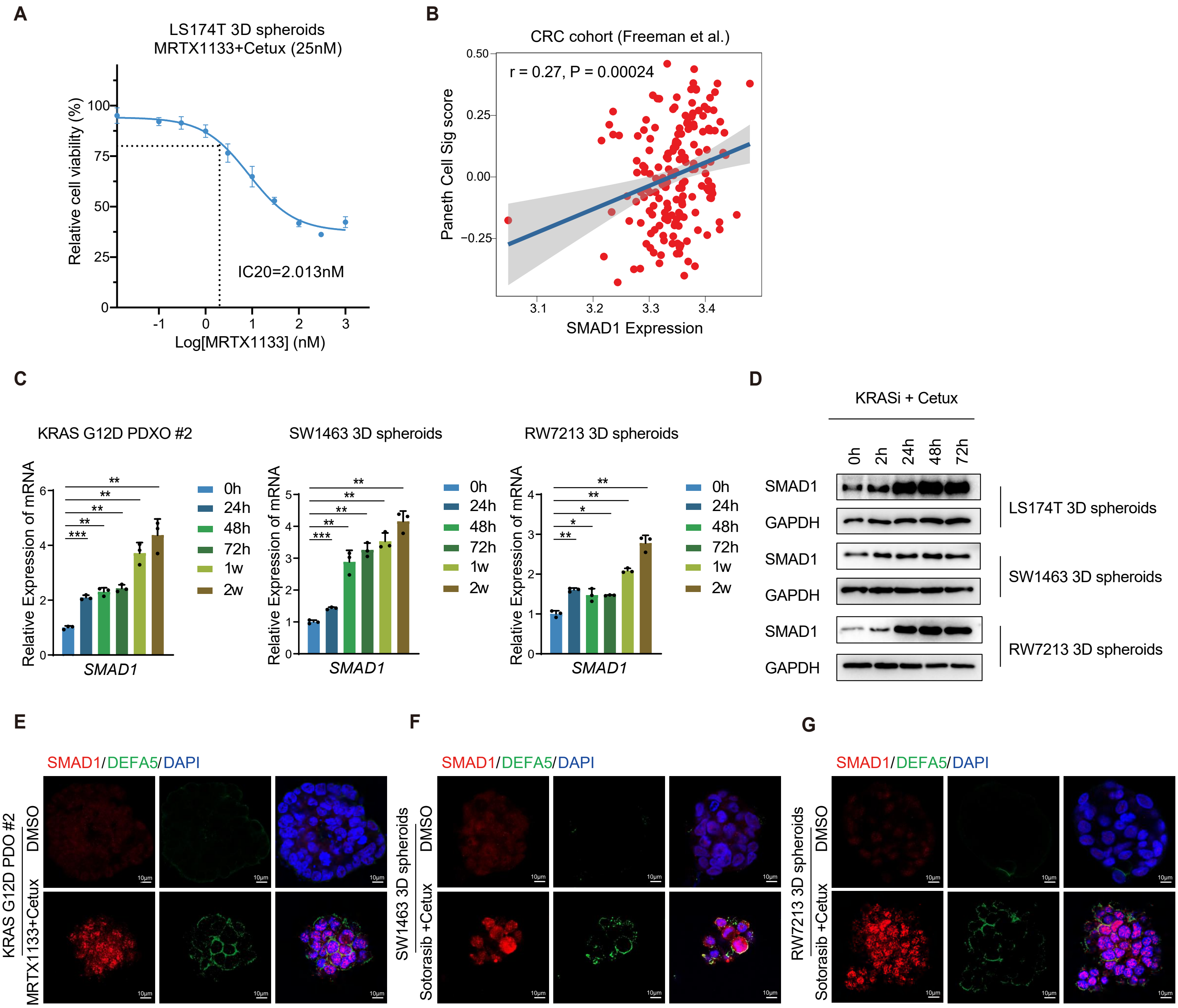

Supplementary Figure 3. CRISPR screening identifies SMAD1 as a Driver of Paneth-like state transition and therapy resistance. Related to Figure 3. (A) Dose–response curve of LS174T 3D spheroids treated with increasing concentrations of MRTX1133 combined with Cetuximab (25nM). Data are represented as mean ± SD, n=6. **(B)** Scatterplots showing the correlation between Paneth cell signature and SMAD1 expression in CRC patients from GSE dataset[48]. Pearson correlation coefficients and p values are shown. **(C)** RT-qPCR analysis of *SMAD1* expression in KRAS G12D PDO#2, SW1463 3D spheroids and RW7213 3D spheroids following a time-course treatment with MRTX1133 or Sotorasib (100nM) + Cetuximab(25nM), normalized to the reference gene *GAPDH*. Data are presented as mean ± SD (n = 3) and analyzed by one-way ANOVA test, *p < 0.05, **p < 0.01, ***p < 0.001. **(D)** Western blot analysis of SMAD1 expression in LS174T 3D spheroids, SW1463 3D spheroids and RW7213 3D spheroids following a time-course treatment with MRTX1133 or Sotorasib (100nM) + Cetuximab (25nM). GAPDH is used as loading control. **(E-G)** Representative immunofluorescence images showing co-expression of DEFA5 and SMAD1 in KRAS G12D PDO#2 (E), SW1463 3D spheroids (F) and RW7213 3D spheroids (G) treated with either DMSO or MRTX1133 or Sotorasib (100nM) + Cetuximab (25nM) for 1w. Scale bar: 10 μm.

Figure S4

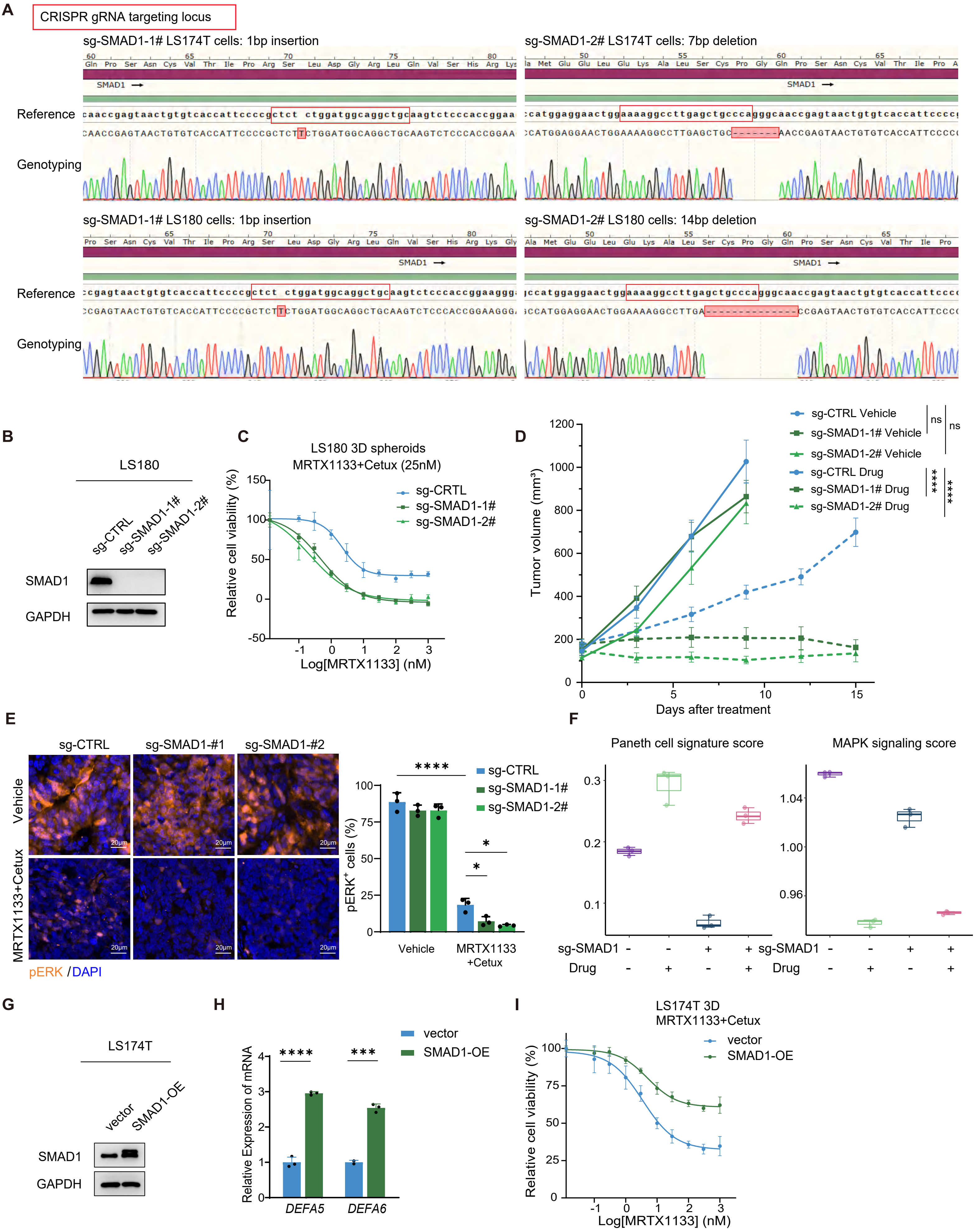
A
CRISPR gRNA targeting locus
sg-SMAD1-1# LS174T cells: 1bp insertion
sg-SMAD1-2# LS174T cells: 7bp deletion
sg-SMAD1-1# LS180 cells: 1bp insertion
sg-SMAD1-2# LS180 cells: 14bp deletion
Reference
Genotyping
B
LS180
sg-CTRL
sg-SMAD1-1#
sg-SMAD1-2#
SMAD1
GAPDH
C
LS180 3D spheroids
MRTX1133+Cetux (25nM)
sg-CRTL
sg-SMAD1-1#
sg-SMAD1-2#
Relative cell viability (%)
Log[MRTX1133] (nM)
D
sg-CTRL Vehicle
sg-SMAD1-1# Vehicle
sg-SMAD1-2# Vehicle
sg-CTRL Drug
sg-SMAD1-1# Drug
sg-SMAD1-2# Drug
ns
****
Tumor volume (mm³)
Days after treatment
E
sg-CTRL
sg-SMAD1-#1
sg-SMAD1-#2
Vehicle
MRTX1133+Cetux
pERK / DAPI
pERK⁺ cells (%)
Vehicle
MRTX1133 +Cetux
F
Paneth cell signature score
MAPK signaling score
sg-SMAD1
Drug
G
LS174T
vector
SMAD1-OE
SMAD1
GAPDH
H
vector
SMAD1-OE
Relative Expression of mRNA
DEFA5
DEFA6
I
LS174T 3D
MRTX1133+Cetux
vector
SMAD1-OE
Relative cell viability (%)
Log[MRTX1133] (nM)

Supplementary Figure 4. SMAD1 drives resistance to KRAS-EGFR combination therapy by promoting Paneth-like state transition. Related to Figure 4. (A) Sequencing results of genotyping in *SMAD1* knockout cell lines. **(B)** Western blot of SMAD1 in LS180 control (sg-CTRL) and *SMAD1* knockout cells generated using two different guide RNAs (sg-SMAD1-1#, sg-SMAD1-2#). GAPDH was used as a loading control. **(C)** Cell viability analysis of LS180 sg-CTRL and *SMAD1* knockout cells following treatment with increasing concentrations of MRTX1133 plus Cetuximab(25nM). Data are presented as mean ± SD, n=6. **(D)** Tumor growth curves comparing LS174T control (sg-CTRL) and *SMAD1* knockout (sg-SMAD1-1#, sg-SMAD1-2#) xenografts over time with or without MRTX1133+Cetux treatment. Data are presented as mean ± SEM (n = 10 per group). Statistical analysis was performed using repeated measures two-way ANOVA to assess overall growth differences; ****$p < 0.0001$, ns, no significance. **(E)** Immunofluorescence staining of pERK in xenograft tumors described in (D) (left), with corresponding quantification (right). Scale bar = 20μm. Comparison was done using one-way ANOVA test, with data presented as mean ± SD (n=3). *$P < 0.05$, ****$P < 0.0001$. **(F)** Boxplots show ssGSEA scores for Paneth cell and MAPK signaling signatures in bulk RNA-seq data from LS174T sg-CTRL and sg-SMAD1 cells treated with DMSO or MRTX1133 + cetuximab for 72 h (n = 3 per group). Boxplots represent median ± interquartile range (IQR), whiskers indicate 1.53 IQR. **(G)** Western blot showing SMAD1 expression in LS174T cells stably transduced with SMAD1 or control vector. GAPDH was used as a loading control. **(H)** RT-qPCR analysis of Paneth cell marker genes *DEFA5* and *DEFA6* in SMAD1-overexpressing cells compared to control vector, normalized to the reference gene *GAPDH*. Statistical significance was determined by two-tailed Student's t-test (n=3); ***$p < 0.001$, ****$p < 0.0001$. **(I)** Cell viability of SMAD1-overexpressing or control LS174T 3D spheroids treated with MRTX1133 plus Cetuximab. Data are shown as mean ± SD, n=6.

Figure S5

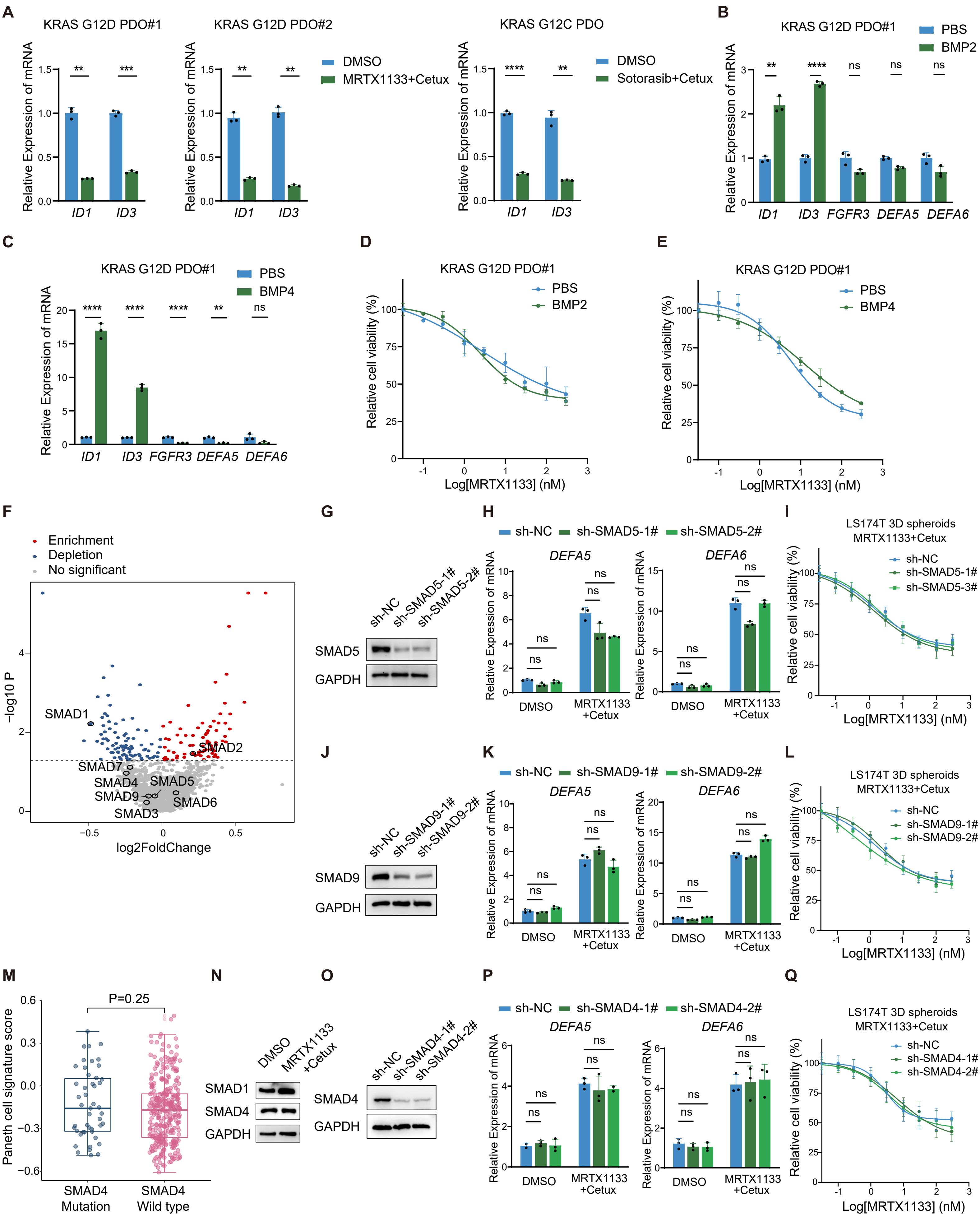

A
KRAS G12D PDO#1
KRAS G12D PDO#2
KRAS G12C PDO
DMSO
MRTX1133+Cetux
Sotorasib+Cetux
Relative Expression of mRNA
ID1
ID3
B
KRAS G12D PDO#1
PBS
BMP2
FGFR3
DEFA5
DEFA6
C
BMP4
D
KRAS G12D PDO#1
Relative cell viability (%)
Log[MRTX1133] (nM)
E
F
Enrichment
Depletion
No significant
−log10 P
log2FoldChange
SMAD1
SMAD2
SMAD7
SMAD4
SMAD5
SMAD9
SMAD6
SMAD3
G
sh-NC
sh-SMAD5-1#
sh-SMAD5-2#
SMAD5
GAPDH
H
DMSO
MRTX1133 +Cetux
I
LS174T 3D spheroids MRTX1133+Cetux
sh-SMAD5-3#
J
sh-SMAD9-1#
sh-SMAD9-2#
SMAD9
K
L
M
P=0.25
Paneth cell signature score
SMAD4 Mutation
SMAD4 Wild type
N
SMAD1
SMAD4
O
sh-SMAD4-1#
sh-SMAD4-2#
P
Q

Supplementary Figure 5. SMAD1 Drives Therapy-Induced Paneth-like Transition Independent of Canonical BMP Signaling and SMAD4. Related to Figure 4. (A) RT-qPCR analysis of BMP signaling output genes (*ID1*, *ID3*) expression in KRAS G12D PDO#1, KRAS G12D PDO#2, and KRAS G12C PDO treated with DMSO and MRTX1133 or Sotorasib + Cetuximab for 1 week, normalized to the reference gene *GAPDH*. Data are presented as mean ± SD (n = 3), two-tailed t-test, $^{**}p < 0.01$, $^{***}p < 0.001$, $^{****}p < 0.0001$. **(B-C)** RT-qPCR analysis of BMP signaling output genes, *FGFR3*, *DEFA5* and *DEFA6* expression in KRAS G12D PDO#1 treated with PBS or BMP2 (100ng/ml), BMP4 (100ng/ml), normalized to the reference gene *GAPDH*. Data are presented as mean ± SD, n = 3, two-tailed t-test, $^{**}p < 0.01$, $^{****}p < 0.0001$, ns, no significance. **(D-E)** Cell viability analysis of KRAS G12D PDO#1 treated with MRTX1133 + Cetuximab, with or without BMP2 (100ng/ml) or BMP4 (100ng/ml). Data were represented as mean ± SD, n = 3. **(F)** Volcano plot illustrating CRISPR screen results highlighting SMAD family members. Genes significantly depleted are shown in blue, enriched genes in red, and those without statistical significance in gray. **(G, J, O)** Western blots confirming knockdown of *SMAD5* (G), *SMAD9* (J), and *SMAD4* (O) in LS174T cells using two shRNAs. GAPDH serves as a loading control. (**H, K, P)** RT-qPCR analysis of Paneth cell markers *DEFA5* and *DEFA6* in control and SMAD knockdown LS174T cells treated with DMSO or MRTX1133 (100 nM) + Cetuximab (25 nM), normalized to the reference gene *GAPDH*. Data are mean ± SD (n = 3), analyzed by one-way ANOVA test, ns, no significance. **(I, L, Q)** Cell viability of control and SMAD knockdown LS174T cells treated with increasing concentrations of MRTX1133 + Cetuximab (25 nM). Data are mean ± SD (n = 6). **(M)** Boxplot showing no significant difference in Paneth cell gene signatures expression between *SMAD4* mutant and wild-type CRCs from TCGA colorectal cancer cohorts[54]. Statistical analysis was performed using the Wilcoxon rank-sum test. Boxplots represent median ± interquartile range (IQR), whiskers indicate 1.53 IQR. **(N)** Western blot analysis of SMAD1 and SMAD4 expression in LS174T 3D spheroids treated with DMSO or MRTX1133 (100nM) + Cetuximab (25nM) for 72 h. GAPDH was used as a loading control.

Figure S6

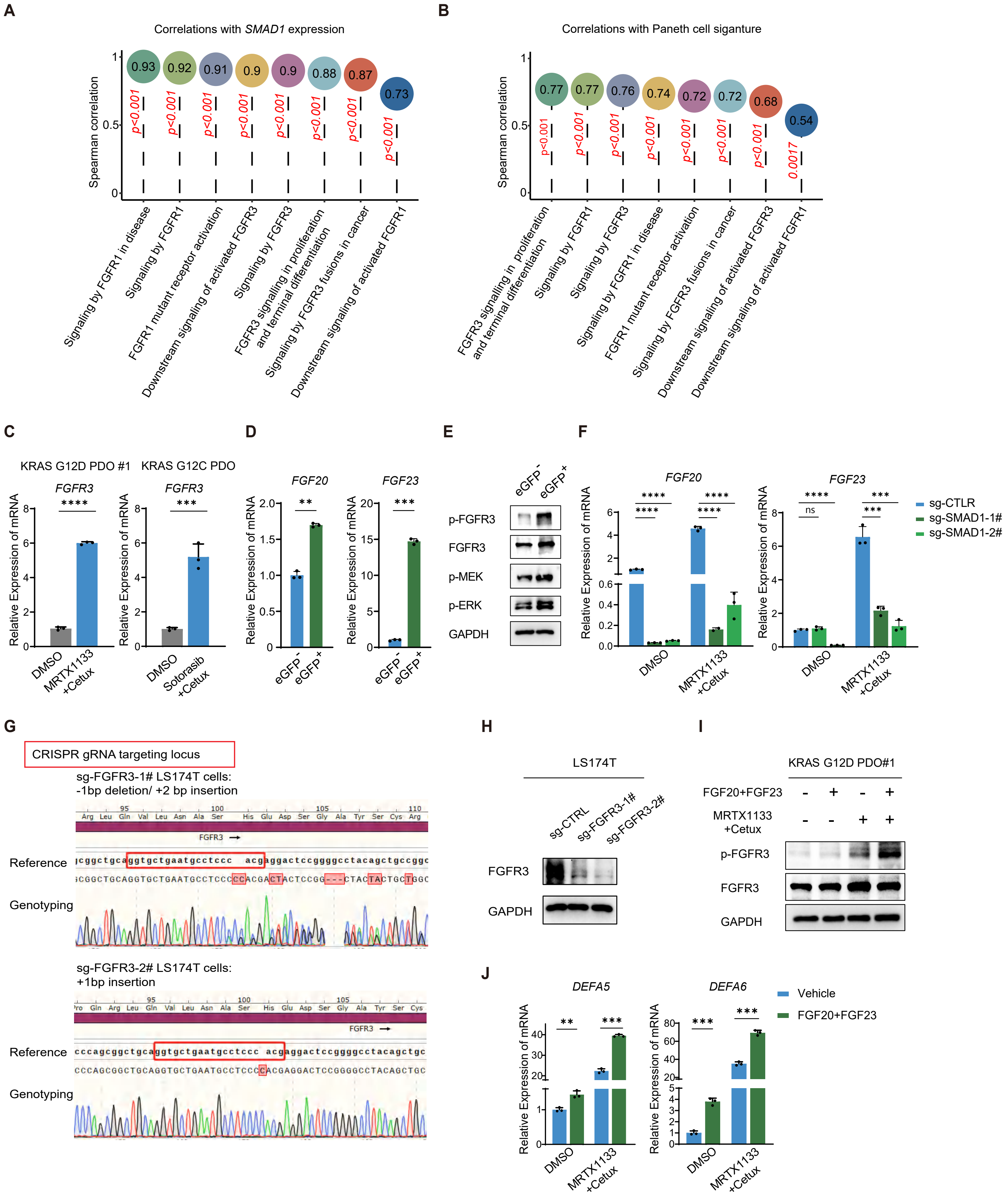

A
Correlations with SMAD1 expression
Spearman correlation
0.93
0.92
0.91
0.9
0.9
0.88
0.87
0.73
Signaling by FGFR1 in disease
Signaling by FGFR1
FGFR1 mutant receptor activation
Downstream signaling of activated FGFR3
Signaling by FGFR3
FGFR3 signalling in proliferation and terminal differentiation
Signaling by FGFR3 fusions in cancer
Downstream signaling of activated FGFR1
B
Correlations with Paneth cell siganture
0.77
0.77
0.76
0.74
0.72
0.72
0.68
0.54
C
KRAS G12D PDO #1
KRAS G12C PDO
FGFR3
Relative Expression of mRNA
DMSO
MRTX1133 +Cetux
Sotorasib +Cetux
D
FGF20
FGF23
eGFP-
eGFP+
E
p-FGFR3
FGFR3
p-MEK
p-ERK
GAPDH
F
sg-CTLR
sg-SMAD1-1#
sg-SMAD1-2#
G
CRISPR gRNA targeting locus
sg-FGFR3-1# LS174T cells:
-1bp deletion/ +2 bp insertion
Reference
Genotyping
sg-FGFR3-2# LS174T cells:
+1bp insertion
H
LS174T
sg-CTRL
sg-FGFR3-1#
sg-FGFR3-2#
I
KRAS G12D PDO#1
FGF20+FGF23
MRTX1133 +Cetux
J
DEFA5
DEFA6
Vehicle
FGF20+FGF23

Supplementary Figure 6. FGFR3 mediates Paneth-like state transition of CRC cells downstream of SMAD1. Related to Figure 5. (A-B) Lollipop charts showing the correlations between SMAD1 (A), Paneth cell signature (B), and FGFR1 and FGFR3 related pathways in publicly available human CRC datasets[48]. Pearson correlation coefficients and p values are shown. **(C)** RT-qPCR analysis of *FGFR3* mRNA expression in KRAS G12D PDO#1 and KRAS G12C PDO treated with DMSO or MRTX1133 or Sotorasib (100nM) +Cetuximab (25nM) for 1 wk, normalized to the reference gene *GAPDH*. Data are presented as mean ± SD (n = 3), analyzed by two-tailed t-test, $***p < 0.001$, $****p < 0.0001$. **(D)** RT-qPCR analysis of *FGF20* and *FGF23* mRNA expression in sorted $eGFP^+$ Paneth-like CRC cells compared with $eGFP^-$ cells, normalized to the reference gene *GAPDH*. Data are presented as mean ± SD (n = 3), analyzed by two-tailed t-test, $**p < 0.01$, $***p < 0.001$. **(E)** Immunoblot analysis of FGFR3, p-FGFR3 (Tyr724), p-MEK, and p-ERK in $eGFP^+$ Paneth-like and $eGFP^-$ cells. GAPDH was used as a loading control.**(F)** RT-qPCR analysis of *FGF20* and *FGF23* mRNA expression in LS174T control (sg-CTRL) and *SMAD1* knockout cells (sg-SMAD1-1#, sg-SMAD1-2#) treated with DMSO or MRTX1133 (100 nM) + Cetuximab (25 nM), normalized to the reference gene *GAPDH*. Data are presented as mean ± SD (n = 3), analyzed by Bonferroni-corrected multiple t-tests, $***p < 0.001$, $****p < 0.0001$. **(G)** Sequencing results of genotyping in *FGFR3* knockout LS174T cell lines. **(H)** Western blot of FGFR3 in LS174T control (sg-CTRL) and *FGFR3* knockout cells (sg-FGFR3-1#, sg-FGFR3-2#). GAPDH was used as a loading control. **(I)** Immunoblot analysis of total and phosphorylated FGFR3 (Tyr724) expression in KRAS G12D PDO#1 treated with DMSO or MRTX1133 (100nM) + Cetuximab (25nM) combined with vehicle or FGF20 (100ng/ml) and FGF23 (100ng/ml). GAPDH was used as a loading control. **(J)** RT-qPCR analysis of Paneth cell marker genes *DEFA5* and *DEFA6* in KRAS G12D PDO#1 samples described in (I), normalized to the reference gene *GAPDH*. Data are presented as mean ± SD (n = 3), analyzed by one-way ANOVA , $**p < 0.01$, $***p < 0.001$.

Figure S7

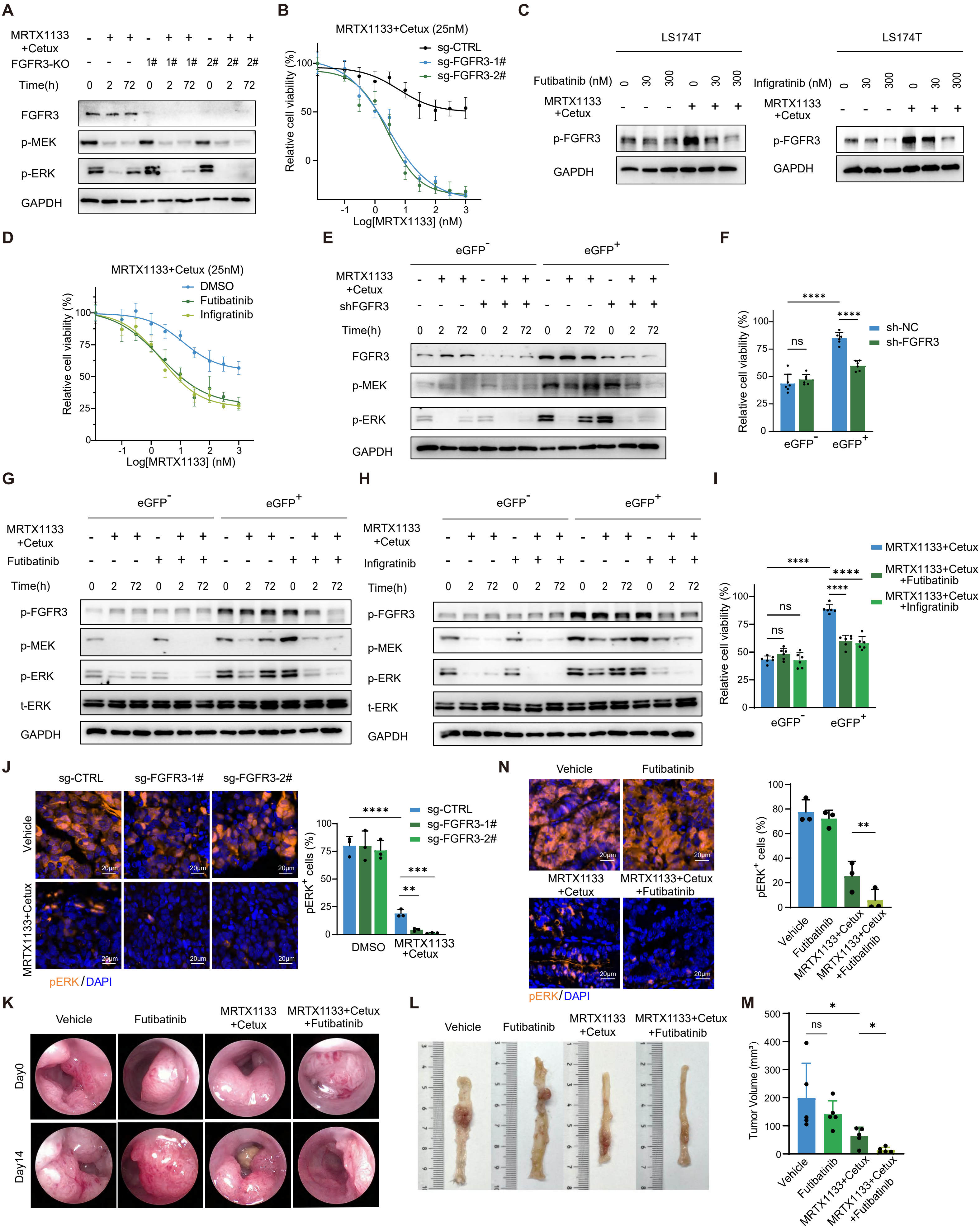
A
MRTX1133 +Cetux
FGFR3-KO
Time(h)
FGFR3
p-MEK
p-ERK
GAPDH
B
MRTX1133+Cetux (25nM)
sg-CTRL
sg-FGFR3-1#
sg-FGFR3-2#
Relative cell viability (%)
Log[MRTX1133] (nM)
C
LS174T
Futibatinib (nM)
Infigratinib (nM)
p-FGFR3
GAPDH
D
DMSO
Futibatinib
Infigratinib
E
eGFP-
eGFP+
shFGFR3
F
sh-NC
sh-FGFR3
G
t-ERK
H
I
MRTX1133+Cetux +Futibatinib
MRTX1133+Cetux +Infigratinib
J
Vehicle
pERK/DAPI
pERK+ cells (%)
DMSO
N
K
Day0
Day14
L
M
Tumor Volume (mm3)

Supplementary Figure 7. FGFR3 promotes MAPK reactivation and therapy resistance in Paneth-like CRC cells. Related to Figure 5. **(A)** Western blot of FGFR3, p-MEK and p-ERK expression in LS174T control (sg-CTRL) and *FGFR3* knockout cells (sg-FGFR3-1#, sg-FGFR3-2#) treated with MRTX1133 (100nM) + Cetuximab (25nM) over time. GAPDH was used as a loading control. **(B)** Cell viability analysis of LS174T control (sg-CTRL) and *FGFR3* knockout cells (sg-FGFR3-1#, sg-FGFR3-2#) treated with MRTX1133 + Cetuximab (25nM). Data were represented as mean ± SD, n=6. **(C)** Western blot of p-FGFR3 (Tyr724) in LS174T 3D spheroids treated with DMSO or MRTX1133 (100nM) + Cetuximab (25nM), combined with various concentrations of Futibatinib (left) and Infigratinib (right), for 72 h. GAPDH was used as a loading control. **(D)** Cell viability analysis of LS174T 3D spheroids treated with MRTX1133 + Cetuximab (25nM), with or without Infigratinib (300nM) and Futibatinib (300nM). Data were represented as mean ± SD, n=6. **(E)** Immunoblot analysis of FGFR3, p-MEK, and p-ERK in *FGFR3* knockdown $eGFP^+$ Paneth-like and $eGFP^-$ cells treated with MRTX1133 (100 nM) + Cetuximab (25 nM) over a time course. GAPDH was used as a loading control. **(F)** Cell viability of *FGFR3* knockdown $eGFP^+$ Paneth-like and $eGFP^-$ cells following treatment with MRTX1133 (300 nM) + Cetuximab (25 nM). Statistical analysis was conducted using one-way ANOVA , with data presented as mean ± SD (n=6). ****p < 0.0001. ns, no significance. **(G-H)** Immunoblot analysis of p-FGFR3 (Tyr724), p-MEK, p-ERK, and total ERK in $eGFP^+$ and $eGFP^-$ cells treated with MRTX1133 (100 nM) + Cetuximab (25 nM) in combination with Futibatinib (300 nM) (G) or Infigratinib (300 nM) (H) over time. GAPDH was used as a loading control. **(I)** Cell viability of $eGFP^+$ and $eGFP^-$ cells treated with MRTX1133 (300 nM) + Cetuximab (25 nM) with or without Futibatinib (300 nM) or Infigratinib (300 nM). Significance was determined using one-way ANOVA, with data presented as mean ± SD (n=6). ****p < 0.0001. ns, no significance. **(J)** Immunofluorescence staining of pERK in *FGFR3* knockout xenografts treated with or without MRTX1133 + Cetuximab (left), and corresponding quantification (right). Scale bar: 20 μm. Data are presented as mean ± SD (n = 3), analyzed by one-way ANOVA, **p < 0.01, ***p < 0.001, ****p < 0.0001. **(K-L)** Representative endoscopic (K) and tumor images (L) of iKAP tumors at Day0 and Day14 in vehicle, Futibatinib, MRTX1133 + Cetuximab, and MRTX1133 + Cetuximab + Futibatinib treatment groups. **(M)** Quantification of tumor volume in each treatment group. Data are presented as mean ± SD (n = 5), analyzed by one-way ANOVA, *p < 0.05. **(N)** Immunofluorescence staining and quantification of pERK in iKAP tumors from (K–M). Scale bar = 20 μm. Data are presented as mean ± SD (n = 3), analyzed by one-way ANOVA test, **p < 0.01.

Figure S8

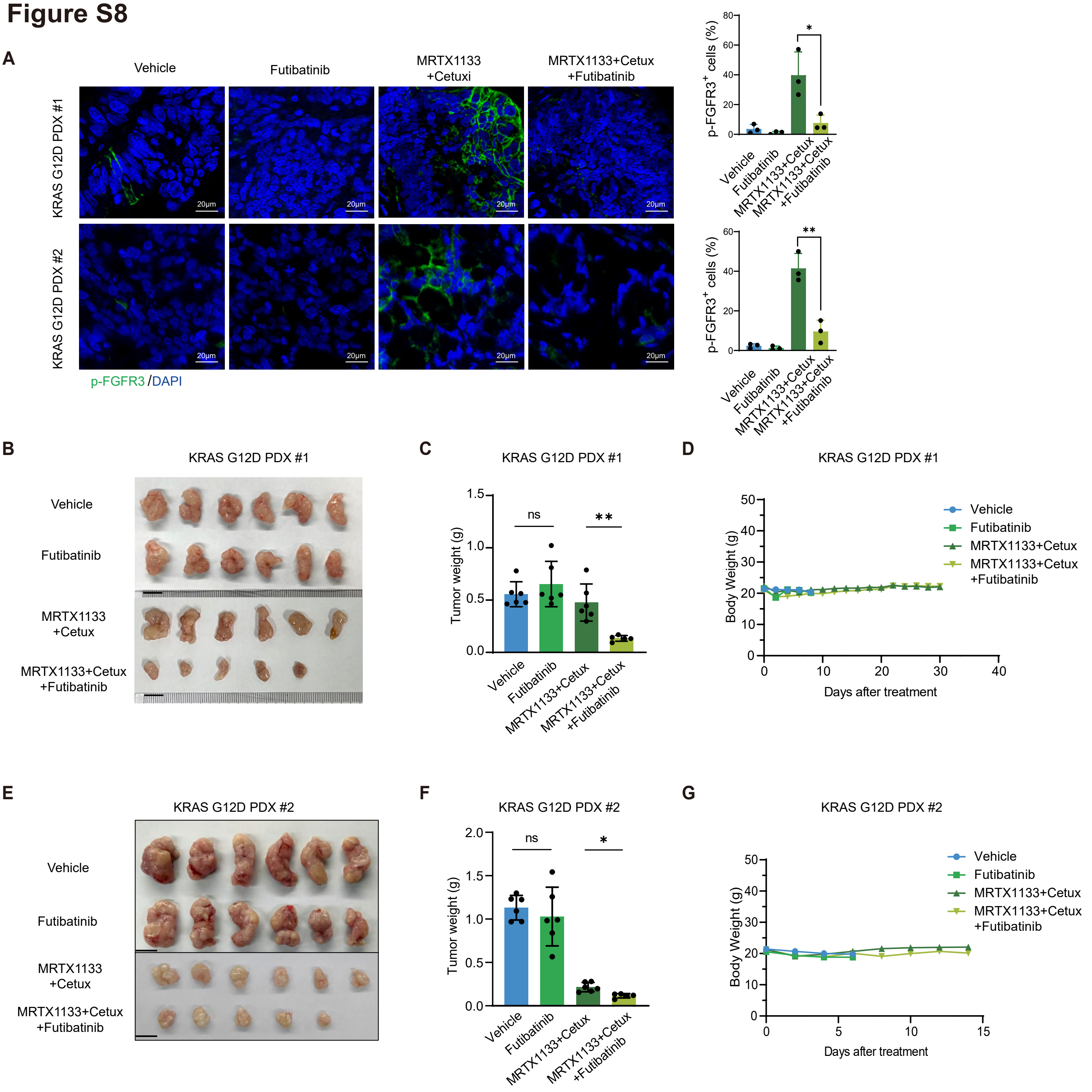

Supplementary Figure 8. Paneth-like state is enriched in human residual CRC tumors following combined KRAS-EGFR inhibition. Related to Figure 6. (A) Immunofluorescence staining of p-FGFR3 (Tyr724) in KRAS G12D PDX#1 (upper) and KRAS G12D PDX#2 (lower) treated with Vehicle, Futibatinib, MRTX1133+Cetux, and triple combination, and corresponding quantification. Scale bar: 20 μm. Data are presented as mean ± SD (n = 3), analyzed by two-tailed Student's t-test, *p < 0.05, **p < 0.01. **(B-D)** Tumor images (B), tumor weight (C) and body weight changes (D) in KRAS G12D CRC PDX#1 model treated with vehicle, Futibatinib, MRTX1133+Cetuximab, or the triple combination for the indicated times. Scale bar in (B), 1cm. Data are presented as mean ± SD (n = 5-6), analyzed by two-tailed Student's t-test, **p < 0.01, ns, no significance. **(E-G)** Tumor images (E), tumor weight (F) and body weight changes (G) in KRAS G12D CRC PDX#2 model treated with vehicle, Futibatinib, MRTX1133+Cetuximab, or the triple combination for the indicated times. Scale bar in (E), 1cm. Data are presented as mean ± SD (n = 5-6), analyzed by two-tailed Student's t-test, *p < 0.05, ns, no significance.

Table S1: Comparison of somatic mutations in pre-treatment and post-treatment samples. Related to Figure 4.

Sample	Patient #1 SYSUCC		Patient #2 MSKCC		KRAS G12D PDX#1		LS174T cells	
**Tumor type**	pre-treatment	post-treatment	pre-treatment	post-treatment	Vehicle-treated	KRAS/EGFR inhibition treated	parental cells	eGFP+ Paneth like cells
**KRAS**	p.G12C	p.G12C	p.G12C	p.G12C	p.G12D	p.G12D	p.G12D	p.G12D
**SMAD4**	---	---	---	---	---	---	---	---
**TP53**	p.V217_Y220 delindD	p.V217_Y220 delindD	---	---	p.Y126H	p.Y126H	p.G360E	p.G360E
**Wnt/β-catenin mutation**	APC p.R405*<br>APC p.Q879*<br>APC p.T1556Nfs*3	APC p.R405*<br>APC p.Q879*<br>APC p.T1556Nfs*3	APC p.R1450*<br>AMER1 p.R353*	APC p.R1450*<br>AMER1 p.R353*	TCF4 p.S767A	TCF4 p.S767A	CTNNB1 p.S45F<br>RNF43 p.K108E<br>RNF43 p.R389H	CTNNB1 p.S45F<br>RNF43 p.K108E<br>RNF43 p.R389H
**Other RTK/ MAPK/PI3K mutation**	AKT2 p.A195T	AKT2 p.A195T	MAP2K4 p.R134W<br>MAP2K4 p.P326L<br>PIK3R1 p.X279_splice	MAP2K4 p.R134W<br>MAP2K4 p.P326L<br>PIK3R1 p.X279_splice	NF1 p.K1385R<br>PIK3CA p.E542K	NF1 p.K1385R<br>PIK3CA p.E542K	PIK3CA p.H1047R	PIK3CA p.H1047R

Table S2. Clinical characteristics of the KRAS mutant CRC patients treated with KRAS inhibitor combined with EGFR antibody. Related to Figure 6 and STAR Methods.

<table>
<tr><td colspan="3">Patient ID</td><td>Patient #1 SYSUCC</td><td>Patient #2 MSKCC</td></tr>
<tr><td colspan="3">Age</td><td>48</td><td>60</td></tr>
<tr><td colspan="3">Sex</td><td>Female</td><td>Male</td></tr>
<tr><td colspan="3">Race</td><td>Asian</td><td>White</td></tr>
<tr><td colspan="3">Diagnosis</td><td>CRC</td><td>CRC</td></tr>
<tr><td colspan="3">Tumor stage</td><td>T4N1M1 IV</td><td>T3N2M1 IV</td></tr>
<tr><td colspan="3">Treatment</td><td>KRAS G12Ci+Cetuximab</td><td>KRAS G12Ci+Panitumumab</td></tr>
<tr><td colspan="3">Date of first dose</td><td>2023/1/18</td><td>2021/4/13</td></tr>
<tr><td rowspan="5">Biopsy samples</td><td rowspan="2">Pre-treatment</td><td>Date</td><td>2022/8/24</td><td>2021/4/12</td></tr>
<tr><td>Type</td><td>Primary tumor</td><td>Liver metastasis</td></tr>
<tr><td rowspan="3">Post-treatment</td><td>Date</td><td>2024/2/20</td><td>2021/12/18</td></tr>
<tr><td>Type</td><td>Liver metastasis</td><td>Peritoneal metastasis</td></tr>
<tr><td>Status</td><td>Progress</td><td>Responding (stable after initial decrease)</td></tr>
</table>

Table S3. Oligonucleotides used in lineage tracing using CRISPR/CAS9 based gene editing strategy, related to STAR Methods.

Primer name	Sequence	Purpose
hDEFA5-sgRNA-target	5'-AGCGACAGCAGAGTCTGTAG-3'	gRNA for base editing
HDR-part1-F	5'-ATTTTATCGATTTCTAGAGCTAGCGAATTCAGCATGCACAATGCGGCATT-3'	HDR donor plasmid construct
HDR-part1-R	5'-CGCTCTCGTCGCTCTCCATGCGACAGCAGAGTCTGTAGAGTCGGCC-3'	HDR donor plasmid construct
HDR-part1-F	5'-ACTCTACAGACTCTGCTGTCGCATGGAGAGCGACGAGAGCG-3'	HDR donor plasmid construct
HDR-part1-R	5'-CTGCTTTGGTTTCTATCTAGGAAGCTCAGCGAGATCCGGTGGAGCC-3'	HDR donor plasmid construct
HDR-part1-F	5'-GGCTCCACCGGATCTCGCTGAGCTTCCTAGATAGAAACCAAAGCAGT-3'	HDR donor plasmid construct
HDR-part1-R	5'-ATCGCAGATCCTTCGCGGCCGCGGATCCATACAAACCATTAGTAGGGTGTGGTGG-3'	HDR donor plasmid construct
hDEFA5-GT-F	5'-GTGCCTGACCCTCTCTTCTT-3'	genotype
hDEFA5-GT-R	5'-AAGGAACCATACAAACCACCA-3'	genotype

Table S4: sgRNAs and shRNAs primers used in this study. Related to STAR Methods.

**sgRNAs**	**forward**
sg-SMAD1-1#-forward	5'-CACCGGCAGCCTGCCATCCAGAGAG-3'
sg-SMAD1-1#-reverse	5'-AAACCTCTCTGGATGGCAGGCTGCC-3'
sg-SMAD1-2#-forward	5'-CACCGAAAAGGCCTTGAGCTGCCCA-3'
sg-SMAD1-2#-reverse	5'-AAACTGGGCAGCTCAAGGCCTTTTC-3'
sg-FGFR3-forward	5'-CACCGGGTGCTGAATGCCTCCCACG-3'
sg-FGFR3-reverse	5'-AAACCGTGGGAGGCATTCAGCACCC-3'
sh-SMAD5-1#-forward	5'- GATCCCCTCAGATTATGCCCAGTATATTCAAGAGATATA CTGGGCATAATCTGAGGTTTTTG -3'
sh-SMAD5-1#-reverse	5'- AATTCAAAAACCTCAGATTATGCCCAGTATATCTCTTGA ATATACTGGGCATAATCTGAGGG -3'
sh-SMAD5-2#-forward	5'-GATCCGCCTAAACATTGGTGTTCAATTTCAAGAGAATT GAACACCAATGTTTAGGCTTTTTG-3'
sh-SMAD5-2#-reverse	5'-AATTCAAAAAGCCTAAACATTGGTGTTCAATTCTCTTGA AATTGAACACCAATGTTTAGGCG-3'
sh-SMAD9-1#-forward	5'-GATCCGCAGAAAGAAGTGTGCATTAATTCAAGAGATTAA TGCACACTTCTTTCTGCTTTTTG-3'
sh-SMAD9-1#-reverse	5'-AATTCAAAAAGCAGAAAGAAGTGTGCATTAATCTCTTGA ATTAATGCACACTTCTTTCTGCG-3'
sh-SMAD9-2#-forward	5'-GATCCCCCTATCAACACTCAGACTTTTTCAAGAGAAAAG TCTGAGTGTTGATAGGGTTTTTG-3'
sh-SMAD9-2#-reverse	5'-AATTCAAAAACCCTATCAACACTCAGACTTTTCTCTTGAA AAAGTCTGAGTGTTGATAGGGG-3'
sh-SMAD4-1#-forward	5'- GATCCCGAGTTGTATCACCTGGAATTTTCAAGAGAAAT TCCAGGTGATACAACTCGTTTTTG -3'
sh-SMAD4-1#-reverse	5'- AATTCAAAAACGAGTTGTATCACCTGGAATTTCTCTTGA AAATTCCAGGTGATACAACTCGG -3'
sh-SMAD4-2#-forward	5'- GATCCGTACTTCATACCATGCCGATTTTCAAGAGAAATC GGCATGGTATGAAGTACTTTTTG -3'
sh-SMAD4-2#-reverse	5'- AATTCAAAAAGTACTTCATACCATGCCGATTTCTCTTGAA AATCGGCATGGTATGAAGTACG -3'

Table S5: Oligonucleotides (qPCR primer sequences) used in this study, related to STAR Methods.

Gene	forward	reverse
GAPDH	5'-AATGAAGGGGTCATTGATGG-3'	5'-AAGGTGAAGGTCGGAGTCAA-3'
DEFA5	5'-GGCTACAACCCAGAAGCAGT-3'	5'-CGGCCACTGATTTCACACAC-3'
DEFA6	5'-CAGGACTTTGCCGTCTCCTT-3'	5'-CATGACAGTGCAGGTCCCAT-3'
LYZ	5'-GCAAAACCCCAGGAGCAGTT-3'	5'-TTGTGGATCACGGACAACCC-3'
SPRY2	5'-GCTGATGGCATAATCCGGGT-3'	5'-AGTCAATCACGTTCTGGGCC-3'
SPRY4	5'-ATTGGATCTCCTTCAGCGGG-3'	5'-AGGCCTCGCACAGCAAGAA-3'
ETV4	5'-TGGAAATCAGGAACAAACTGC-3'	5'-GCCCCTCGACTCTGAAGAT-3'
DUSP6	5'-CGAATCTCTTTGAGAACGCAGG-3'	5'-TGTCATAGGCATCGTTCATCG-3'
SMAD1	5'-TACCCTCACTCTCCCACCAG-3'	5'-TTGTGGAGGAGGCATGGAAC-3'
ID1	5'-GGTGCGCTGTCTGTCTGAG-3'	5'-TGTCGTAGAGCAGCACGTTT-3'
ID3	5'-AAATCCTACAGCGCGTCATC-3'	5'-AAGCTCCTTTTGTCGTTGGA-3'
FGFR3	5'-GGAGAACAGCGCCTACAGTAT-3'	5'-CTCCACAAACTCGCACTCGG-3'
FGF20	5'-AGCCTCTTCGGTATCTTGGAAT-3'	5'-TCTTCAAACTGCTCCCTAAAGA-3'
FGF23	5'-GCACCCCATCAGACCATCTA-3'	5'-GTCTGGTGTTGGAACCTGCA-3'
FGFR3-promoter-primer1	5'-AGCTAAGCCGCCTGAGAGC-3'	5'-TGGAGCCCAGAACCTCGGG-3'
FGFR3-promoter-primer2	5'-TGGGTTCCCAGGAGGGGAA-3'	5'-AAGCCACAAGCCAGGCAC-3'
